# Multiscale Modelling of De Novo Anaerobic Granulation

**DOI:** 10.1007/s11538-021-00951-y

**Published:** 2021-11-06

**Authors:** A. Tenore, F. Russo, M. R. Mattei, B. D’Acunto, G. Collins, L. Frunzo

**Affiliations:** 1 Department of Mathematics and Applications, University of Naples “Federico II”, Via Cintia 1, Monte S’ Angelo, 80126 Naples, Italy; 2 Microbial Communities Laboratory, School of Biological and Chemical Sciences, National University of Ireland Galway, University Road, Galway, H91 TK33 Ireland

**Keywords:** Biofilm, Free boundary value problem, Spherical symmetry, Granulation, Anaerobic digestion

## Abstract

A multiscale mathematical model is presented to describe de novo granulation, and the evolution of multispecies granular biofilms, in a continuously fed bioreactor. The granule is modelled as a spherical free boundary domain with radial symmetry. The equation governing the free boundary is derived from global mass balance considerations and takes into account the growth of sessile biomass as well as exchange fluxes with the bulk liquid. Starting from a vanishing initial value, the expansion of the free boundary is initiated by the attachment process, which depends on the microbial species concentrations within the bulk liquid and their specific attachment velocity. Nonlinear hyperbolic PDEs model the growth of the sessile microbial species, while quasi-linear parabolic PDEs govern the dynamics of substrates and invading species within the granular biofilm. Nonlinear ODEs govern the evolution of soluble substrates and planktonic biomass within the bulk liquid. The model is applied to an anaerobic, granular-based bioreactor system, and solved numerically to test its qualitative behaviour and explore the main aspects of de novo anaerobic granulation: ecology, biomass distribution, relative abundance, dimensional evolution of the granules and soluble substrates, and planktonic biomass dynamics within the bioreactor. The numerical results confirm that the model accurately describes the ecology and the concentrically layered structure of anaerobic granules observed experimentally, and that it can predict the effects on the process of significant factors, such as influent wastewater composition; granulation properties of planktonic biomass; biomass density; detachment intensity; and number of granules.

## Introduction

Biofilms are complex, dense and compact aggregates comprising microbial cells immobilized in a self-produced matrix of extracellular polymeric substances (EPS) (Flemming and Wingender 2010). Many species from several trophic groups may coexist in such structures, where they interact through synergistic and antagonistic activities. Although natural biofilms typically develop as planar layers attached to suitable surfaces, under specific conditions the aggregation occurs due to the self-immobilization of cells into approximately spherical-shaped granules (Trego et al. 2020a). The process leading to the formation of these aggregates is known as granulation. In particular, the term de novo granulation is used when the process is initiated by individual microbial cells and flocs, as opposed to when granulation proceeds from inocula already in a granular form.

In recent years, granular bioreactor systems have become increasingly popular in the field of sustainable, and high-rate, wastewater treatment. Compared to suspended biomass systems, the denser, stronger and more regular structure of the biofilm granules underpins better settling properties (Liu and Tay 2002, 2004) allowing for higher concentrations of biomass (Baeten et al. 2019) and reduced bioreactor footprints (Liu and Tay 2002). Furthermore, and in contrast to other biofilm systems in which the biofilms develop on solid supports, granular systems are based on spherical, and constantly moving, microbial aggregates. The movement and shape mitigate boundary layer resistances and enhance the mass transfer of substrates across the biofilm granule (Baeten et al. 2019). For these reasons, granular biofilms have been successfully developed in different bioreactor configurations, for various processes, such as aerobic, anaerobic and partial nitritation-anammox treatments (Trego et al. 2020b).

The main drawback of granular-based systems is represented by the start-up phase, due to the complexity of the mechanisms and phenomena which contribute to the success of the granulation process (Li et al. 2015). Many studies have explored the granulation process and numerous theories have been proposed. Hydrodynamic conditions generated by liquid up-flow velocity, gas production, particle–particle collision, mixing systems and bioreactor geometry are universally recognized as key factors in the granulation process as well as throughout the entire life cycle of the granules (Liu and Tay 2002; Trego et al. 2020b; Lettinga et al. 1980). Indeed, suitable hydrodynamic conditions are required to initiate the granulation process by promoting, and improving, the aggregation of planktonic biomass (Lemaire et al. 2008). Moreover, intense hydrodynamic conditions induce high shear forces on the granule surface, influencing size, shape, structure and density of the granules (Liu and Tay 2002; Di Iaconi et al. 2006), and regulate a continuous process of aggregation and breaking that leads to the formation of an increasing number of granules. High shear forces are thought to stimulate the production of EPS, which represents a further beneficial factor for granulation as it increases cell surface hydrophobicity (Trego et al. 2020b). Several studies also consider the granulation process to be the result of an organized process driven by pioneering microbial species with specific, key characteristics (Pol et al. 2004; Wiegant 1988).

Pol et al. (2004) collated various theories concerning anaerobic sludge granulation, the most widespread of which asserts that the process is favoured by key microorganisms, such as *Methanosaeta* (Trego et al. 2020b; Pol et al. 2004; Jian and Shi-yi 1993; Fang 2000). Such acetoclastic methanogens have filamentous structures and good adhering properties and initiate the granulation process by forming a central nucleus supporting the immobilization of other methanogens and synergistically functioning bacterial groups (MacLeod et al. 1990; Zheng et al. 2006). In this context, various studies (Li et al. 2015; Zhang et al. 2012; Li et al. 2014) report that quorum sensing plays an essential role by regulating the transition of some *Methanosaeta* species from short to long, filamentous cells. In the initial phase, the nucleus presents a filamentous appearance and achieves a spherical shape due to the rolling effect of the hydraulic shear forces (Trego et al. 2020b; Pol et al. 2004). In a second phase, the nucleus develops into a granule, and acetogens and acidogens attach on its surface to grow syntrophically with acetoclastic methanogens (Trego et al. 2020b; Pol et al. 2004; MacLeod et al. 1990; Vanderhaegen et al. 1992). The result is a concentrically layered structure with an archaeal core constituted by *Methanosaeta*. This theory is supported by experimental evidence showing layered structures in anaerobic granules (Sekiguchi et al. 1999; Batstone et al. 2004; Collins et al. 2005). Nonetheless, the granulation process is still not fully understood and further studies are required to assist in optimizing the efficiencies of this process.

In this framework, mathematical modelling represents a valuable tool to describe, explore and study the granulation process, the life cycle of the biofilm granules and the performances of granular-based bioreactor systems. The relevance of those topics in environmental engineering and biotechnology has stimulated interest in modelling of granular biofilm systems. Indeed, numerous models have been proposed to mainly describe aerobic (De Kreuk et al. 2007), anaerobic (Batstone et al. 2004; Odriozola et al. 2016; Feldman et al. 2017; Doloman et al. 2017) and anammox (Volcke et al. 2010, 2012; Vangsgaard et al. 2012) processes involved in such systems. An initial classification may be introduced according to the approach used: continuum models simulate the evolution of the granular biofilm in a quantitative and deterministic way, while discrete models, such as individual-based (Doloman et al. 2017; Picioreanu et al. 2005; Xavier et al. 2007) and cellular automata models (Skiadas and Ahring 2002), can represent the multidimensional structural heterogeneity of granular biofilms but provide results including elements of randomness and introduce stochastic effects into the solutions (Mattei et al. 2018). Most models of granular biofilms (Batstone et al. 2004; Odriozola et al. 2016; Feldman et al. 2017; Volcke et al. 2010, 2012) are based on the continuum approach introduced by Wanner and Gujer (1986) for one-dimensional planar biofilms and model the granule as a spherical, free boundary domain evolved as a result of the prevailing microbial metabolic processes and mass exchange with the surrounding environment. Among these, most describe the dynamic evolution of the granule fixing the final steady-state size (Batstone et al. 2004; Feldman et al. 2017; Volcke et al. 2010, 2012).

In any case, several significant aspects of granular biofilm growth are not exhaustively considered by existing models. According to Baeten et al. (2019), only two models (Batstone et al. 2004; Seok and Komisar 2003) consider the attachment process, which plays a key role in the formation and evolution of granular biofilms. None takes into account the invasion process, i.e. the colonization of a pre-existing biofilm mediated by motile planktonic cells living in the surrounding environment, which can penetrate the porous matrix of the biofilm and convert to sessile biomass. Moreover, all continuum models fix a nonzero initial size of the domain and this requires the composition of the initial domain to be arbitrarily fixed. Finally, according to the exclusion principle presented in Klapper and Szomolay (2011), all biofilm models based on the approach introduced in Wanner and Gujer (1986) lead to restrictions on ecological structure.

Most studies have focused on system performance by describing the biofilm-mediated removal of soluble substrates from wastewater. Some focus on the biofilm granule, paying attention to the dimensional evolution (Odriozola et al. 2016) and to the distribution of sessile biomass within the biofilm at the steady-state (Odriozola et al. 2016; Feldman et al. 2017; Volcke et al. 2010, 2012; Vangsgaard et al. 2012). However, no continuous model fully describes the de novo granulation process by considering the initial formation and ecology of the biofilm granule. Only the individual-based model introduced by Doloman et al. (2017) focuses on the de novo formation of anaerobic granules based on a discrete approach.

In this work, we propose a multiscale model to describe the de novo granulation process, and which incorporates the mesoscopic, granular biofilm processes within a continuously fed, granular-based bioreactor. For this purpose, and following the approach proposed by Mašić and Eberl (2012, 2014) in the case of one-dimensional planar biofilms, our model couples macroscopic bioreactor mass balances with a mesoscopic granular biofilm model here derived by using a continuum approach (Wanner and Gujer 1986). The model accounts for the growth of both granular attached and planktonic biomass and includes the main microbial exchange processes involved, including attachment, detachment and invasion. The de novo granulation process is modelled by assuming that all biomass initially present in the bioreactor is in planktonic form. Mathematically, this corresponds to consider a vanishing initial value of the granule radius representing the free boundary under the assumption of radial symmetry. Biofilm formation is initiated by the attachment process, which leads to consider a space-like free boundary. This mathematical problem has been discussed in D’Acunto et al. (2019) and is applied here for the first time to model the genesis of granular biofilms. Granule formation and expansion are governed by the following processes: microbial growth, attachment, invasion and detachment. Attachment initiates the life of biofilms and is regarded as the complex phenomenon whereby pioneering microbial cells in planktonic form attach to a surface and develop in the form of a sessile aggregate (Palmer et al. 2007). However, as reported above, the formation of a biofilm granule is the result of the interaction and aggregation of microbial cells and flocs without the involvement of a surface. Therefore, in this work, attachment is viewed as the flux of microbial mass that aggregates, switches its phenotype from planktonic to sessile and initiates the granulation. It is modelled as a linear function of the concentrations of the planktonic species, each of which is characterized by a specific attachment velocity. The invasion process is included for the first time in the modelling of granular biofilms by extending the mathematical formulation proposed in D’Acunto et al. (2015) for one-dimensional planar biofilms to a spherical domain. This allows removal of the restrictions on ecological structure highlighted by Klapper and Szomolay (2011). Furthermore, the bulk liquid is modelled as a perfectly mixed medium in which soluble substrates and planktonic biomass are found, and which is influenced by the operational parameters of the bioreactor, the microbial metabolic activities and the processes of mass exchange with the biofilm. The mathematical model has been derived for a generic, granular-based bioreactor and applied to the anaerobic granulation process to test the mode’s behaviour and study the genesis, evolution and ecology of anaerobic granules. Various numerical studies have investigated how the granulation properties of planktonic biomass, the biomass density of the granules, the detachment intensity, the number of granules and the composition of the influent wastewater may affect the evolution of the process. The results include the dimensional evolution and ecology of the granule (in terms of biomass distribution and relative abundance), the distribution of soluble substrates within the granule and the time variation of soluble substrates and planktonic biomass within the bioreactor.

The paper is organized as follows: in Sect. 2, the derivation of the model is carried out by presenting all assumptions, variables, equations, and initial and boundary conditions; Sect. 3 then describes the biological case to which the model is applied. Numerical studies are reported, and discussed in detail, in Sects. 4 and 5, respectively. Finally, the conclusions of the work, and future goals, are outlined in Sect. 6.

## Mathematical Model

**Fig. 1:**
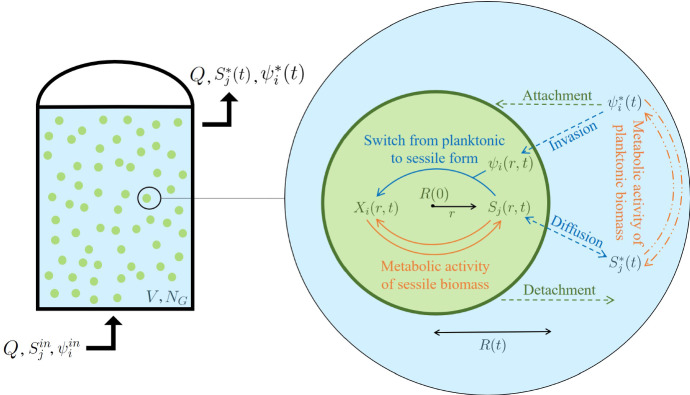
Multiscale representation of the model. The bioreactor is modelled as a perfectly mixed continuous system (on the left), having volume *V*, where $N_G$ biofilm granules are immersed. A focus on a single granule is presented on the right, with all processes considered in the model. The granule has a zero initial radius *R*(0), which varies over time due to the effect of various biological processes. Metabolic processes within the granule are carried out by the sessile biomass $X_i(r,t)$, which grows by converting the substrates dissolved in the biofilm liquid $S_j(r,t)$, while metabolic processes within the bulk liquid are carried out by the planktonic biomasses $\psi ^*_i(t)$, which grows by converting the substrates dissolved in the bulk liquid $S^*_j(t)$. The superficial exchange processes of attachment and detachment are considered at the interface granule-bulk liquid. Moreover, invasion processes are modelled: the planktonic biomass $\psi _i(r,t)$ invades the solid matrix of the granule and switches its phenotype from planktonic to sessile. Finally, process of diffusion of substrates across the granule is included in the model. Solid arrows: processes within the granule. Dash-dot arrows: processes within the bulk liquid. Dash arrows: exchange processes between granule and bulk liquid (color figure online)

In this work, the granular biofilm reactor is modelled as a completely mixed, continuously fed system in which $N_G$ identical biofilm granules are immersed. As shown in Fig. 1, two different scales are considered in the model: the bioreactor macroscale and the granule mesoscale. Three components are considered within the granular biofilm: the sessile biomass, which constitutes the solid matrix; the planktonic biomass, which is found in the channels and voids; and the soluble substrates dissolved in the liquid phase. Meanwhile, planktonic biomass and soluble substrates are considered within the bulk liquid of the reactor. These components interact with, and influence, each other as a result of biological, physical and chemical processes. Modelling of both the granule and bioreactor scales is discussed, and we introduce each of the processes, assumptions, variables, equations and initial and boundary conditions involved.

### Modelling Granule Scale

Under the assumption of radial symmetry, the biofilm granule is modelled as a spherical, free boundary domain whose spatial evolution is completely described by the evolution of the radius *R*(*t*). A vanishing initial value $R(0)=0$ is considered to model the initial granulation. All variables involved in the biofilm modelling are considered as functions of time *t* and space *r*, where *r* denotes the radial coordinate. Consequently, the granule centre is located at $r = 0$.

The model takes into account the dynamics of three components, expressed in terms of concentration: *n* microbial species in sessile form $X_i(r,t)$; *n* microbial species in planktonic form $\psi _i(r,t)$; and *m* dissolved substrates $S_j(r,t)$.

The volume occupied by planktonic cells is considered negligible due to the small particle size. The density of the granule $\rho$ is assumed to be constant and equal for all microbial species. By dividing sessile species concentration $X_i$ by $\rho$, biomass volume fractions $f_i(r,t)$ are achieved. $f_i$ are constrained to add up to unity (Rahman et al. 2015). In summary, the model components describing the granular biofilm compartment are:

1 $$\begin{aligned}&X_i,\ i=1,...,n,\ \mathbf{X}=(X_1,...,X_n), \end{aligned}$$

2 $$\begin{aligned}&f_i=\frac{X_i}{\rho },\ i=1,...,n,\ \mathbf{f}=(f_1,...,f_n), \end{aligned}$$

3 $$\begin{aligned}&\psi _i,\ i=1,...,n,\ {{\varvec{\psi }}}=(\psi _1,...,\psi _n), \end{aligned}$$

4 $$\begin{aligned}&S_j,\ j=1,...,m,\ \mathbf{S}=(S_1,...,S_m). \end{aligned}$$

Based on the continuum approach introduced in Wanner and Gujer (1986) for one-dimensional planar biofilms, a system of partial differential equations (PDEs) in a spherical, free boundary domain is derived from mass balance considerations, under the assumption of radial symmetry. Hyperbolic PDEs model the distribution and growth of sessile biomass $f_i(r,t)$ and parabolic PDEs describe the diffusion and conversion of soluble substrates $S_j(r,t)$. Further parabolic PDEs govern the process of invasion and conversion of planktonic cells $\psi _i(r,t)$.

Based on the aggregation properties of the planktonic biomass living in the bulk liquid, attachment phenomena govern the initial granulation process, while further biofilm evolution is significantly affected by detachment phenomena. Attachment and detachment contribute to the microbial mass exchange occurring between granules and bulk liquid and are included in the model as continuous and deterministic processes. Finally, as introduced by D’Acunto et al. (2015), in the case of one-dimensional planar biofilm, the invasion process is considered to describe the phenomena of granule colonization by planktonic cells. Such cells penetrate the porous matrix of the biofilm from the surrounding medium and contribute to the development of the biofilm.

Under the assumption of radial symmetry, the mass balance set up for a generic component in a differential volume of the spherical domain leads to the following equation:

5 $$\begin{aligned} \frac{\partial c(r,t)}{\partial t} +\frac{1}{r^2} \frac{\partial }{\partial r}(r^2 J_r(r,t)) =r_c(r,t), \end{aligned}$$

where *c*(*r*, *t*) is the concentration of a generic component in the spherical domain, $J_r$ is the advective and/or diffusive flux in the radial direction and $r_c(r,t)$ is the transformation term.

The transport of sessile biomass is modelled as an advective process. Hence, by expressing the advective flux of the *i*th sessile microbial species in the radial direction as

6 $$\begin{aligned} J_{r,i}(r,t)=u(r,t) X_i(r,t), \end{aligned}$$

where *u*(*r*, *t*) is the biomass velocity, Eq. (5) takes the following form:

7 $$\begin{aligned}&\frac{\partial X_i(r,t)}{\partial t} +\frac{1}{r^2} \frac{\partial }{\partial r}(r^2 u(r,t) X_i(r,t)) = \rho r_{M,i}(r,t,\mathbf{X},\mathbf{S}) + \rho r_i(r,t,{\varvec{\psi }},\mathbf{S}), \nonumber \\&\quad \qquad i=1,...,n, 0 \le r \le R(t),\ t>0, \end{aligned}$$

where $r_{M,i}^{}(r,t,\mathbf{X},\mathbf{S})$ and $r_i(r,t,{\varvec{\psi }},\mathbf{S})$ are the specific growth rates due to sessile and planktonic species, respectively.

By dividing Eq. (7) by $\rho$ and by considering that $f_i=\frac{X_i}{\rho }$ yields

8 $$\begin{aligned}&\frac{\partial f_i(r,t)}{\partial t} +\frac{1}{r^2} \frac{\partial }{\partial r}(r^2 u(r,t) f_i(r,t)) =r_{M,i}(r,t,\mathbf{f},\mathbf{S})+r_i(r,t,{\varvec{\psi }},\mathbf{S}), \nonumber \\&\qquad \quad i=1,...,n, 0 \le r \le R(t),\ t>0, \end{aligned}$$

9 $$\begin{aligned}&\frac{\partial f_i(r,t)}{\partial t} + f_i(r,t) \frac{\partial u(r,t)}{\partial r} + \frac{2 u(r,t) f_i(r,t)}{r} + u(r,t) \frac{\partial f_i(r,t)}{\partial r}\nonumber \\&\qquad \quad =r_{M,i}(r,t,\mathbf {f},\mathbf {S}) +r_i(r,t,{\varvec{\psi }},\mathbf {S}),\nonumber \\&\qquad \quad \ i=1,...,n, 0 \le r \le R(t),\ t>0. \end{aligned}$$

Summing Eq. (9) over all sessile microbial species *i* and considering that $\sum _{i=1}^{n}f_i=1$, it follows:

10 $$\begin{aligned} \frac{\partial u(r,t)}{\partial r} = -\frac{2 u(r,t)}{r} + G(r,t,\mathbf {f },\mathbf {S }, {\varvec{\psi }}), 0 < r \le R(t),\ t>0, \end{aligned}$$

where $G(r,t,\mathbf{f },\mathbf{S }, {\varvec{\psi }})=\sum _{i=1}^{n}(r_{M,i}(r,t,\mathbf{f },\mathbf{S })+r_i(r,t,{\varvec{\psi }},\mathbf{S }))$. This differential equation governs the evolution of the biomass velocity *u*(*r*, *t*).

By imposing the flux of the *i*th sessile microbial species equal to 0 at $r=0$, it follows from Eq. (6) that $u(0,t)=0$. Considering this result and integrating Eq. (10), the integral expression of *u*(*r*, *t*) is achieved:

11 $$\begin{aligned} u(r,t)=\frac{1}{r^2}\int _{0}^{r} r'^2 G(r',t,\mathbf{f },\mathbf{S }, {\varvec{\psi }}) \mathrm{d}r', 0 < r \le R(t),\ t>0. \end{aligned}$$

Substituting Eq. (10) into Eq. (9) yields

12 $$\begin{aligned}&\frac{\partial f_i(r,t)}{\partial t} + u(r,t)\frac{\partial f_i(r,t)}{\partial r} =r_{M,i}(r,t,\mathbf {f},\mathbf {S})+r_i(r,t,{\varvec{\psi }},\mathbf {S}) \nonumber \\&\qquad \quad - f_i(r,t) G(r,t,\mathbf {f },\mathbf {S }, {\varvec{\psi }}), \nonumber \\&\qquad \quad i=1,...,n, 0 \le r \le R(t),\ t>0. \end{aligned}$$

Equation (12) describes the transport and growth of the sessile microbial species *i* across the granular biofilm under the assumption of radial symmetry.

Compared to the equation reported in Wanner and Gujer (1986) for a planar biofilm, Eq. (12) presents a different expression of *u*(*r*, *t*) and the additional reaction term $r_i(r,t,{\varvec{\psi }},\mathbf{S})$ due to the invasion phenomenon.

Equation (5) can be applied to soluble substrates and planktonic species. In these cases, the transport of planktonic biomass and soluble substrates is modelled as a diffusive flux and expressed as

13 $$\begin{aligned} J_{r,\psi _i}(r,t)=-D_{\psi ,i}\frac{\partial \psi _i(r,t)}{\partial r}, \end{aligned}$$

and

14 $$\begin{aligned} J_{r,j}(r,t)=-D_{S,j}\frac{\partial S_j(r,t)}{\partial r}, \end{aligned}$$

where $D_{\psi ,i}$ and $D_{S,j}$ denote the diffusivity coefficient of the planktonic species *i* and the soluble substrate *j* in the biofilm, respectively.

Then, parabolic diffusion–reaction PDEs are derived from Eq. (5):

15 $$\begin{aligned}&\frac{\partial \psi _i(r,t)}{\partial t}-D_{\psi ,i}\frac{\partial ^2 \psi _i(r,t)}{\partial r^2} - \frac{2 D_{\psi ,i}}{r} \frac{\partial \psi _i(r,t)}{\partial r}= r_{\psi ,i}(r,t,{\varvec{\psi }},\mathbf{S}), \nonumber \\&\quad \quad \quad \ i=1,...,n, 0< r < R(t),\ t>0, \end{aligned}$$

16 $$\begin{aligned}&\frac{\partial S_j(r,t)}{\partial t}-D_{S,j}\frac{\partial ^2 S_j(r,t)}{\partial r^2} - \frac{2 D_{S,j}}{r} \frac{\partial S_j(r,t)}{\partial r}= r_{S,j}(r,t,\mathbf{f},\mathbf{S}), \nonumber \\&\qquad \quad \ j=1,...,m, 0< r < R(t),\ t>0, \end{aligned}$$

where $r_{\psi ,i}(r,t,{\varvec{\psi }},\mathbf{S})$ is the conversion rate of planktonic species *i* and $r_{S,j}(r,t,\mathbf{f},\mathbf{S})$ is the conversion rate of soluble substrate *j*.

The free boundary evolution is described by the variation of the radius *R*(*t*) over time. This is affected by microbial growth and processes of attachment and detachment occurring at the surface of the biofilm. In particular, as proposed by Wanner and Reichert (1996), the attachment flux of the *i*th planktonic species is formulated as a function linearly dependent on the concentration of the planktonic species *i* in the bulk liquid $\psi ^*_i(t)$ and is expressed as:

17 $$\begin{aligned} \sigma _{a,i}(t)=\frac{v_{a,i}\psi ^*_i(t)}{\rho },\ i=1,...,n, \end{aligned}$$

where $v_{a,i}$ is the attachment velocity of the planktonic species *i*.

By summing Eq. (17) over all planktonic species, the total attachment flux is achieved:

18 $$\begin{aligned} \sigma _a(t)=\frac{\sum _{i=1}^{n}v_{a,i}\psi ^*_i(t)}{\rho }. \end{aligned}$$

The detachment is modelled as a quadratic function of the granule radius *R*(*t*) (Abbas et al. 2012):

19 $$\begin{aligned} \sigma _d(t)=\lambda R^2(t), \end{aligned}$$

where $\lambda$ is the detachment coefficient and is supposed to be equal for all microbial species.

The global mass balance on the spherical domain gives:

20 $$\begin{aligned} \frac{\partial }{\partial t}\int _{0}^{R(t)} 4 \pi r^2 \rho \mathrm{d}r =\rho A(t) (\sigma _a(t)-\sigma _d(t))+ \int _{0}^{R(t)} 4 \pi r^2 \rho G(r,t,\mathbf{f },\mathbf{S }, {\varvec{\psi }}) \mathrm{d}r, \end{aligned}$$

where *A*(*t*) is the area of the spherical granule and is equal to $4 \pi R^2(t)$.

By dividing Eq. (20) by $4 \pi \rho$ and by considering *u*(*R*(*t*), *r*) from Eq. (11), it follows:

21 $$\begin{aligned} \frac{\partial }{\partial t}\int _{0}^{R(t)} r^2 \mathrm{d}r= & {} R^2(t) (\sigma _a(t)-\sigma _d(t))+ \int _{0}^{R(t)} r^2 G(r,t,\mathbf{f },\mathbf{S }, {\varvec{\psi }}) \mathrm{d}r, \end{aligned}$$

22 $$\begin{aligned} \frac{1}{3}\frac{\partial R^3(t)}{\partial t}= & {} R^2(t) (\sigma _a(t)-\sigma _d(t))+ R^2(t) u(R(t),t), \end{aligned}$$

23 $$\begin{aligned} {\dot{R}}(t)= & {} \sigma _a(t)-\sigma _d(t) + u(R(t),t). \end{aligned}$$

The latter equation governs the time evolution of the free boundary domain.

The total mass of the sessile community and the mass of the *i*th sessile microbial species within the granule can be calculated as follows:

24 $$\begin{aligned} m_i(t)= & {} \int _{0}^{R(t)} 4 \pi r^2 \rho f_i(r,t) \mathrm{d}r,\ i=1,...,n, \end{aligned}$$

25 $$\begin{aligned} m_{tot}(t)= & {} \sum _{i=1}^{n} m_i(t) = \frac{4}{3} \pi \rho R^3(t). \end{aligned}$$

### Modelling Reactor Scale

As already mentioned, the reactor is modelled as a completely mixed continuous system. Thus, all the quantities referring to the bulk liquid dynamics are equal at every point and are dependent on time. The variables considered in the bulk liquid are *n* planktonic biomasses and *m* soluble substrates, both expressed in terms of concentration ($\psi ^*_i(t)$ and $S^*_j(t)$, respectively). Such concentrations vary over time due to biological processes, operational parameters of the reactor and mesoscopic granule processes. In summary, the model components, which describe the bulk liquid compartment, are:

26 $$\begin{aligned}&\psi ^*_i,\ i=1,...,n,\ {{\varvec{\psi }}^*}=(\psi ^*_1,...,\psi ^*_n), \end{aligned}$$

27 $$\begin{aligned}&S^*_j,\ j=1,...,m,\ \mathbf{S^*}=(S^*_1,...,S^*_m). \end{aligned}$$

Accordingly, a system of ordinary differential equations (ODEs) derived from mass balance considerations is considered to describe the dynamics of planktonic biomass and soluble substrates within the bulk liquid:

28 $$\begin{aligned} V {{\dot{\psi }}}^*_i(t)=&{} Q(\psi ^{in}_i-\psi ^*_i(t))- A(t) N_G D_{\psi ,i} \frac{\partial \psi _i(R(t),t)}{\partial r}+r^*_{\psi ,i}(t,{{\varvec{\psi }}^*},\mathbf {S^*})+ \nonumber \\&-\sigma _{a,i}(t) \rho A(t) N_G,\ i=1,...,n\,\ t>0, \end{aligned}$$

29 $$\begin{aligned} V {\dot{S}}^*_j(t)=&{} Q(S^{in}_j-S^*_j(t))- A(t) N_G D_{S,j} \frac{\partial S_j(R(t),t)}{\partial r} +r^*_{S,j}(t,{{\varvec{\psi }}^*},\mathbf {S^*}), \nonumber \\&\ j=1,...,m,\,\ t>0. \end{aligned}$$

where *V* is the volume of the bulk liquid assumed equal to the reactor volume, *Q* is the continuous flow rate, $\psi ^{in}_i$ is the concentration of the planktonic species *i* in the influent, $S^{in}_j$ is the concentration of the substrate *j* in the influent, $r^*_{\psi ,i}(t,{{\varvec{\psi }}^*},\mathbf{S^*})$ and $r^*_{S,j}({t,{\varvec{\psi }}^*},\mathbf{S^*})$ are the conversion rates for $\psi _i^*$ and $S_j^*$, respectively.

Equation (28) represents the mass balance of the *i*th microbial species in planktonic form. In particular, the mass variation within the bioreactor (first member) is due to the continuous mass flow in and out of the bioreactor (first term of the second member), the exchange flux between the bulk liquid and the granular biofilms (second term of the second member), the growth and decay in the bulk liquid (third term of the second member), and the exchange flux related to attachment processes (fourth term of the second member).

Similarly, Eq. (29) represents the mass balance of the *j*th soluble substrate. In this case, the mass variation within the bioreactor (first member) is due to the continuous mass flow in and out of the bioreactor (first term of the second member), the exchange flux between the bulk liquid and the granular biofilms (second term of the second member) and the consumption, and/or production, occurring in the bulk liquid and mediated by the planktonic biomass (third term of second member).

### Initial and Boundary Conditions

The processes involved in a granular biofilm reactor are described by Eqs. (10), (12), (15), (16), (23), (28) and (29). To integrate such equations, it is necessary to specify initial and boundary conditions.

As mentioned above, de novo granulation is modelled by considering an initial configuration whereby only planktonic biomass is supposed to be present in the reactor. Hence, a vanishing initial condition is coupled to Eq. (23), which describes the variation of the granule radius over time:

30 $$\begin{aligned} R(0)= 0. \end{aligned}$$

The following initial conditions are considered for Eqs. (28) and (29):

31 $$\begin{aligned} \psi ^*_i(0)= & {} \psi ^*_{i,0}, \ i=1,...,n, \end{aligned}$$

32 $$\begin{aligned} S^*_j(0)= & {} S^*_{j,0},\ j=1,...,m, \end{aligned}$$

where $\psi ^*_{i,0}$ and $S^*_{j,0}$ are the initial concentrations of the *i*th planktonic species and the *j*th soluble substrate within the bulk liquid, respectively.

Equations (10), (12), (15) and (16) refer to the biofilm domain and do not require initial conditions, since the extension of the biofilm domain is zero at $t = 0$.

The boundary condition for Eq. (12) at the interface granule-bulk liquid $r = R(t)$ depends on the sign of the mass flux at the interface. When the free boundary is a space-like line ($\sigma _{a}-\sigma _{d}>0$), there is a mass flux from bulk liquid to biofilm; thus, the boundary condition depends on the concentration of planktonic biomass in the bulk liquid:

33 $$\begin{aligned} f_i(R(t),t) = \frac{v_{a,i}\psi ^*_i(t)}{\sum _{i=1}^{n}v_{a,i}\psi ^*_i(t)}, \ i=1,...,n,\ t>0. \end{aligned}$$

Meanwhile, when the free boundary is a time-like line ($\sigma _{a}-\sigma _{d}<0$), the biomass concentration at the interface is regulated exclusively by the internal points of the biofilm domain and the condition (33) is not required.

For both parabolic systems (15) and (16), a no-flux condition is fixed at the granule centre $r = 0$, while boundary conditions at the interface biofilm-bulk liquid $r = R(t)$ are related to the solutions of Eqs. (28) and (29), which represent the concentrations of planktonic species and soluble substrates within the bulk liquid:

34 $$\begin{aligned}&\frac{\partial \psi _i}{\partial r}(0,t)=0,\ \psi _i(R(t),t))=\psi ^*_i(t),\ i=1,...,n,\ t>0, \end{aligned}$$

35 $$\begin{aligned}&\frac{\partial S_j}{\partial r}(0,t)=0,\ S_j(R(t),t))=S^*_j(t),\ j=1,...,m,\ t>0. \end{aligned}$$

Finally, as previously mentioned, the boundary condition for Eq. (10) is given by:

36 $$\begin{aligned} u(0,t)=0,\ t>0. \end{aligned}$$

In conclusion, the model is based on Eqs. (10), (12), (15), (16), (23), (28), (29), and initial and boundary conditions Eqs. (30)–(36). All equations and initial and boundary conditions are summarized in Table 1. The reaction terms of these equations depend on the specific biological case considered and describe the complex biological interplay taking place between sessile biomass $X_i(r,t)$, planktonic biomass $\psi _i(r,t)$ and soluble substrates $S_j(r,t)$ within the biofilm and planktonic biomass $\psi ^*_i(t)$ and soluble substrates $S^*_j(t)$ within the bulk liquid.

**Table 1:** Model equations and initial and boundary conditions

Equations	Initial condition	Boundary condition	
	$t=0$	$r=0$	$r = R(t)$
$\frac{\partial f_i(r,t)}{\partial t} + u(r,t)\frac{\partial f_i(r,t)}{\partial r} = r_{M,i}(r,t,\mathbf{f},\mathbf{S})+r_i(r,t,{{\varvec{\psi }}},\mathbf{S})- f_i(r,t) G(r,t,\mathbf{f },\mathbf{S }, {\varvec{\psi }}))$			$f_i(R(t),t) = \frac{v_{a,i}\psi ^*_i(t)}{\sum _{i=1}^{n}v_{a,i}\psi ^*_i(t)}$ when $\sigma _{a}-\sigma _{d}>0$
$\frac{\partial \psi _i(r,t)}{\partial t}-D_{\psi ,i}\frac{\partial ^2 \psi _i(r,t)}{\partial r^2} - \frac{2 D_{\psi ,i}}{r} \frac{\partial \psi _i(r,t)}{\partial r}= r_{\psi ,i}(r,t,{{\varvec{\psi }}},\mathbf{S})$		$\frac{\partial \psi _i}{\partial r}(0,t)=0$	$\psi _i(R(t),t))=\psi ^*_i(t)$
$\frac{\partial S_j(r,t)}{\partial t}-D_{S,j}\frac{\partial ^2 S_j(r,t)}{\partial r^2} - \frac{2 D_{S,j}}{r} \frac{\partial S_j(r,t)}{\partial r}=r_{S,j}(r,t,\mathbf{f},\mathbf{S}))$		$\frac{\partial S_j}{\partial r}(0,t)=0$	$S_j(R(t),t))=S^*_j(t)$
$\frac{\partial u(r,t)}{\partial r} = -\frac{2 u(r,t)}{r} + G(r,t,\mathbf{f },\mathbf{S }, {\varvec{\psi }})$		$u(0,t)=0$	
${\dot{R}}(t)= \sigma _a(t)-\sigma _d(t)+u(R(t),t)$	$R(0)= 0$		
$V {{\dot{\psi }}}^*_i(t)=Q(\psi ^{in}_i-\psi ^*_i(t))- A(t) N_G D_{\psi ,i} \frac{\partial \psi _i(R(t),t)}{\partial r}+r^*_{\psi ,i}(t,{{\varvec{\psi }}^*},\mathbf{S^*})-\sigma _{a,i}(t) \rho A(t) N_G$	$\psi ^*_i(0)=\psi ^*_{i,0}$		
$V {\dot{S}}^*_j(t)=Q(S^{in}_j-S^*_j(t))- A(t) N_G D_{S,j} \frac{\partial S_j(R(t),t)}{\partial r}+r^*_{S,j}(t,{{\varvec{\psi }}^*},\mathbf{S^*})$	$S^*_j(0)=S^*_{j,0}$		

## Modelling de novo Anaerobic Granulation

The mathematical model described in the previous section can be applied to any granular biofilm system by defining appropriate variables, parameters, and initial, and boundary, conditions and reaction terms based on the biological processes involved.

In this work, the model is applied to study the process of de novo granulation and the ecology of granules in an anaerobic bioreactor. Anaerobic digestion (AD) is a biological process extensively used to manage liquid and solid wastes, and to produce renewable biofuels. AD underpins low-cost environmental biotechnologies underpinned by a complex, multi-step process in which different trophic groups of microbial species convert organic matter to methane-rich biogas. Over the past few decades, the application of AD has been developed in granular biofilm systems, in which the microbial community forms dense biofilm granules offering several operational advantages over conventional, suspended biomass systems (Baeten et al. 2019; Liu and Tay 2002).

Many studies report the fundamental role played by methanogenic species, which facilitate the formation of the granule nucleus (Trego et al. 2020b; Li et al. 2014, 2015; Zhang et al. 2012). To model this aspect, different attachment velocities are used depending on the microbial species.

The following variables, expressed in terms of concentrations, are included in the model:

- Five sessile microbial components: sugar fermenters $X_{Su}$, butyrate consumers $X_{Bu}$, propionate consumers $X_{Pro}$, acetoclastic methanogens $X_{Ac}$ and inert material $X_{I}$.
- Four planktonic species within the biofilm: sugar fermenters $\psi _{Su}$, butyrate consumers $\psi _{Bu}$, propionate consumers $\psi _{Pro}$ and acetoclastic methanogens $\psi _{Ac}$.
- Five soluble compounds within the biofilm: sugar $S_{Su}$, butyrate $S_{Bu}$, propionate $S_{Pro}$, acetate $S_{Ac}$ and methane $S_{CH_4}$.
- Four planktonic species within the bulk liquid: sugar fermenters $\psi ^*_{Su}$, butyrate consumers $\psi ^*_{Bu}$, propionate consumers $\psi ^*_{Pro}$ and acetoclastic methanogens $\psi ^*_{Ac}$.
- Five soluble compounds within the bulk liquid: sugar $S^*_{Su}$, butyrate $S^*_{Bu}$, propionate $S^*_{Pro}$, acetate $S^*_{Ac}$ and methane $S^*_{CH_4}$.

Inert material is not considered in the bulk liquid as it is supposed to play no role in the life cycle of the granular biofilm (inactive biomass is supposed to have neither metabolic activity nor granulation or invasion properties.)

The model considers an influent flow comprised exclusively of dissolved substrates. Therefore, disintegration and hydrolysis processes, which lead to the conversion of organic matter into soluble compounds, are neglected. The main intracellular processes are taken into account both in the biofilm and in the bulk liquid: acidogenesis, acetogenesis and methanogenesis. The kinetic expressions of the biological processes involved in the model are taken from Batstone et al. (2002). In particular, each growth process leads to the formation of new biomass, and consumption and/or production of one or more soluble substrates. Each decay process implies the death of active biomass, which becomes inert material. Within the biofilm, sessile sugar fermenters $X_{Su}$ grow by converting sugar $S_{Su}$ into butyrate $S_{Bu}$, propionate $S_{Pro}$ and acetate $S_{Ac}$ (i.e. acidogenesis). Butyrate $S_{Bu}$ and propionate $S_{Pro}$ are consumed by sessile butyrate consumers $X_{Bu}$ and sessile propionate consumers $X_{Pro}$, respectively, and acetate $S_{Ac}$ is produced (i.e. acetogenesis). Lastly, acetate is converted into methane $S_{CH_4}$ by sessile acetoclastic methanogens $X_{Ac}$ (i.e. methanogenesis). The same biological processes are supposed to occur in the bulk liquid, in which the planktonic biomass $\psi ^*_i$ consume or produce the soluble substrates $S^*_j$. Furthermore, the decay of any sessile biomass is considered to produce inert material $X_I$, which represents inactive biomass and accumulates in the biofilm. The decay processes are also considered for planktonic species in the bulk liquid.

The planktonic active species present in the bulk liquid are also modelled in the granule domain as planktonic cells $\psi _i$, which populate the voids of the solid matrix and contribute to the growth of the corresponding sessile species as a result of invasion phenomena.

Each of the reaction terms of the model equations are listed below. The specific growth rates within the biofilm due to sessile biomass $r_{M,i}$ in Eqs. (10) and (12) are modelled as Monod-type kinetics:

37 $$\begin{aligned} r_{M,i} = f_{i} \left( \mu _{\max ,i} \frac{S_{i}}{K_{i}+S_{i}}-k_{d,i}\right) , \ i \in I_B, \end{aligned}$$

where $I_B = \{Su, Bu, Pro, Ac\}$ is the index set, $\mu _{\max ,i}$ is the maximum net growth rate for biomass *i*, $K_i$ is the affinity constant of the consumed substrate for biomass *i* and $k_{d,i}$ is the decay constant for biomass *i*.

The inert formation rate is given by the sum of the decay rates of each active species, modelled as first order kinetic:

38 $$\begin{aligned} r_{M,I} = \sum _{i \in I_B} f_{i} \ k_{d,i}. \end{aligned}$$

The specific growth rates within the biofilm due to planktonic cells $r_i$ in Eqs. (10) and (12) are defined as:

39 $$\begin{aligned} r_{i} = k_{col,i} \ \frac{\psi _{i}}{\rho } \frac{S_{i}}{K_{i}+S_{i}}, \ i \in I_B, \end{aligned}$$

where $k_{col,i}$ is the maximum colonization rate of motile species *i* and ${\rho }$ is the granule density.

The conversion rates for planktonic cells due to the invasion process $r_{\psi ,i}$ in Eq. (15) are expressed by:

40 $$\begin{aligned} r_{\psi ,i} = -\frac{1}{Y_{\psi ,i}}r_{i} \ \rho , \ i \in I_B, \end{aligned}$$

where $Y_{\psi ,i}$ denotes the yield of non-motile species *i* on corresponding motile species.

The conversion rates for soluble substrates within the biofilm $r_{S,j}$ in Eq. (16), with $j \in \{Su,Bu,Pro,Ac,CH_4\}$, are listed below:

41 $$\begin{aligned} r_{S,Su}= & {} - \frac{\mu _{\max ,Su}}{Y_{Su}} \frac{S_{Su}}{K_{Su} + S_{Su}}f_{Su} \ \rho , \end{aligned}$$

42 $$\begin{aligned} r_{S,{Bu}}=&{} - \frac{\mu _{\max ,{Bu}}}{Y_{Bu}} \frac{S_{Bu}}{K_{{Bu}} +S_{Bu}}f_{Bu} \ \rho \ \nonumber \\&+ \ g_{Su,Bu} \frac{(1-Y_{Su})}{Y_{Su}} \mu _{\max ,Su} \frac{S_{Su}}{K_{Su}+S_{Su}}f_{Su} \ \rho , \end{aligned}$$

43 $$\begin{aligned} r_{S,{Pro}}=&{} - \frac{\mu _{\max ,{Pro}}}{Y_{Pro}} \frac{S_{Pro}}{K_{{Pro}}+S_{Pro}}f_{Pro} \ \rho \ \nonumber \\&+ \ g_{Su,Pro} \frac{(1-Y_{Su})}{Y_{Su}} \mu _{\max ,Su} \frac{S_{Su}}{K_{Su}+S_{Su}}f_{Su} \ \rho , \end{aligned}$$

44 $$\begin{aligned} r_{S,{Ac}}=&{} -\frac{\mu _{\max ,{Ac}}}{Y_{Ac}} \frac{S_{Ac}}{K_{{Ac}}+S_{Ac}}f_{Ac} \ \rho \ \nonumber \\&+ \ g_{Su,{Ac}} \frac{(1-Y_{Su})}{Y_{Su}}\mu _{\max ,Su} \frac{S_{Su}}{K_{Su}+S_{Su}} f_{Su} \ \rho \ \nonumber \\&+ g_{Bu,Ac} \frac{(1-Y_{Bu})}{Y_{Bu}} \mu _{\max ,{Bu}} \frac{S_{Bu}}{K_{{Bu}}+S_{Bu}}f_{Bu} \ \rho \ \nonumber \\&+ \ g_{Pro,Ac} \frac{(1-Y_{Pro})}{Y_{Pro}} \mu _{\max ,{Pro}} \frac{S_{Pro}}{K_{{Pro}}+S_{Pro}}f_{Su} \ \rho , \end{aligned}$$

45 $$\begin{aligned} r_{S,{CH_4}}= & {} \frac{(1-Y_{Ac})}{Y_{Ac}} \mu _{\max ,{Ac}} \frac{S_{Ac}}{K_{{Ac}}+S_{Ac}}f_{Ac} \ \rho , \end{aligned}$$

where $Y_{Su}$, $Y_{Bu}$, $Y_{Pro}$, $Y_{Ac}$, denote the yields of sugar fermenters, butyrate consumers, propionate consumers and acetoclastic methanogens on the corresponding substrate consumed, $g_{Su,Bu}$, $g_{Su,Pro}$, $g_{Su,Ac}$ are the stoichiometric fractions of butyrate, propionate and acetate produced from sugar, $g_{Bu,Ac}$ and $g_{Pro,Ac}$ are the stoichiometric fractions of acetate produced from butyrate and propionate.

Moreover, the conversion rates of planktonic biomasses $r^*_{\psi ,i}$ within the bulk liquid in Eq. (28) are defined as:

46 $$\begin{aligned} r^*_{\psi ,i} = \psi _{i}^* \left( \mu _{\max ,i} \frac{S_{i}^*}{K_{i}+S_{i}^*}-k_{d,i}\right) , \ i \in I_B, \end{aligned}$$

while the conversion rates of soluble substrates $r^*_{S,j}$ within the bulk liquid in Eq. (29), with $j \in \{Su,Bu,Pro,Ac,CH_4 \}$, are listed below:

47 $$\begin{aligned} r^*_{S,Su}= & {} -\psi _{Su}^* \frac{\mu _{\max ,Su}}{Y_{Su}} \frac{S_{Su}^*}{K_{Su}+S_{Su}^*}, \end{aligned}$$

48 $$\begin{aligned} r^*_{S,{Bu}}=&{} -\psi _{Bu}^* \frac{\mu _{\max ,{Bu}}}{Y_{Bu}} \frac{S_{Bu}^*}{K_{{Bu}}+S_{Bu}^*} \nonumber \\&+ g_{Su,Bu} \frac{(1-Y_{Su})}{Y_{Su}} \psi _{Su}^* \mu _{\max ,Su} \frac{S_{Su}^*}{K_{Su}+S_{Su}^*} , \end{aligned}$$

49 $$\begin{aligned} r^*_{S,{Pro}}=&{} -\psi _{Pro}^* \frac{\mu _{\max ,{Pro}}}{Y_{Pro}} \frac{S_{Pro}^*}{K_{{Pro}}+S_{Pro}^*} \ \nonumber \\&+ \ g_{Su,Pro} \frac{(1-Y_{Su})}{Y_{Su}} \psi _{Su}^* \mu _{\max ,Su} \frac{S_{Su}^*}{K_{Su}+S_{Su}^*}, \end{aligned}$$

50 $$\begin{aligned} r^*_{S,{Ac}}=&{} -\psi _{Ac}^* \frac{\mu _{\max ,{Ac}}}{Y_{Ac}} \frac{S_{Ac}^*}{K_{{Ac}}+S_{Ac}^*} \nonumber \\&+ g_{Su,Ac} \frac{(1-Y_{Su})}{Y_{Su}} \psi _{Su}^* \mu _{\max ,Su} \frac{S_{Su}^*}{K_{Su}+S_{Su}^*} \ \nonumber \\&+ g_{Bu,Ac} \frac{(1-Y_{Bu})}{Y_{Bu}} \psi _{Bu}^* \mu _{\max ,{Bu}} \frac{S_{Bu}^*}{K_{{Bu}}+S_{Bu}^*} \ \nonumber \\&+ \ g_{Pro,Ac} \frac{(1-Y_{Pro})}{Y_{Pro}} \psi _{Pro}^* \mu _{\max ,{Pro}} \frac{S_{Pro}^*}{K_{{Pro}}+S_{Pro}^*}, \end{aligned}$$

51 $$\begin{aligned} r^*_{S,{CH_4}}= & {} \frac{(1-Y_{Ac})}{Y_{Ac}} \psi _{Ac}^* \mu _{\max ,{Ac}} \frac{S_{Ac}^*}{K_{{Ac}}+S_{Ac}^*}. \end{aligned}$$

The values used for all stoichiometric and kinetic parameters are reported in Table 2.

**Table 2:** Kinetic, stoichiometric and operating parameters used for numerical simulations

Parameter	Definition	Unit	Value	Ref
$\mu _{max,Su}$	Maximum specific growth rate for sugar fermenters	$d^{-1}$	3	(a)
$\mu _{max,Bu}$	Maximum specific growth rate for butyrate consumers	$d^{-1}$	1.2	(a)
$\mu _{max,Pro}$	Maximum specific growth rate for propionate consumers	$d^{-1}$	0.52	(a)
$\mu _{max,Ac}$	Maximum specific growth rate for acetoclastic methanogens	$d^{-1}$	0.4	(a)
$k_{d,Su}$	Decay-inactivation rate for sugar fermenters	$d^{-1}$	0.02	(a)
$k_{d,Bu}$	Decay-inactivation rate for butyrate consumers	$d^{-1}$	0.02	(a)
$k_{d,Pro}$	Decay-inactivation rate for propionate consumers	$d^{-1}$	0.02	(a)
$k_{d,Ac}$	Decay-inactivation rate for acetoclastic methanogens	$d^{-1}$	0.02	(a)
$K_{Su}$	Sugar half saturation constant sugar fermenters	$gCOD \ \mathrm {m}^{-3}$	500	(a)
$K_{Bu}$	Butyrate half saturation constant butyrate consumers	$gCOD \ \mathrm {m}^{-3}$	300	(a)
$K_{Pro}$	Propionate half saturation constant propionate consumers	$gCOD \ \mathrm {m}^{-3}$	300	(a)
$K_{Ac}$	Acetate half saturation constant acetoclastic methanogens	$gCOD \ \mathrm {m}^{-3}$	150	(a)
$Y_{Su}$	Yield of sugar fermenters on sugar	$-$ $-$	0.10	(a)
$Y_{Bu}$	Yield of butyrate consumers on butyrate	$-$ $-$	0.06	(a)
$Y_{Pro}$	Yield of propionate consumers on propionate	$-$ $-$	0.04	(a)
$Y_{Ac}$	Yield of acetoclastic methanogens on acetate	$-$ $-$	0.05	(a)
$g_{Su,Bu}$	Fraction of butyrate from sugar	$-$ $-$	0.13	(a)
$g_{Su,Pro}$	Fraction of propionate from sugar	$-$ $-$	0.27	(a)
$g_{Su,Ac}$	Fraction of acetate from sugar	$-$ $-$	0.41	(a)
$g_{Bu,Ac}$	Fraction of acetate from butyrate	$-$ $-$	0.80	(a)
$g_{Pro,Ac}$	Fraction of acetate from propionate	$-$ $-$	0.57	(a)
$D_{S,Su}$	Diffusion coefficient of sugar in biofilm	$m^2 \ d^{-1}$	$4.63\cdot 10^{-5}$	(b)
$D_{S,Bu}$	Diffusion coefficient of butyrate in biofilm	$m^2 \ d^{-1}$	$6.01\cdot 10^{-5}$	(b)
$D_{S,Pro}$	Diffusion coefficient of propionate in biofilm	$m^2 \ d^{-1}$	$7.33\cdot 10^{-5}$	(b)
$D_{S,Ac}$	Diffusion coefficient of acetate in biofilm	$m^2 \ d^{-1}$	$8.36\cdot 10^{-5}$	(b)
$D_{S,CH_4}$	Diffusion coefficient of methane in biofilm	$m^2 \ d^{-1}$	$10.3\cdot 10^{-5}$	(b)
$k_{col,i}$	Maximum colonization rate of *i*th planktonic species	$d^{-1}$	0.001	(c)
$Y_{\psi ,i}$	Yield of non-motile microorganisms on motile species	$-$ $-$	0.001	(c)
$D_{\psi ,i}$	Diffusion coefficient of *i*th planktonic species in biofilm	$m^2 \ d^{-1}$	$10^{-5}$	(c)
$v_{a,Su}$	Attachment velocity of planktonic species $\psi _{Su}$	$m \ d^{-1}$	$3\cdot 10^{-3}$	(c)
$v_{a,Bu}$	Attachment velocity of planktonic species $\psi _{Bu}$	$m \ d^{-1}$	$3\cdot 10^{-3}$	(c)
$v_{a,Pro}$	Attachment velocity of planktonic species $\psi _{Pro}$	$m \ d^{-1}$	$3\cdot 10^{-3}$	(c)
$v_{a,Ac}$	Attachment velocity of planktonic species $\psi _{Ac}$	$m \ d^{-1}$	$150\cdot 10^{-3}$	(c)
$\rho$	Biofilm density	$gCOD \,\mathrm{m}^{-3}$	120000	(c)
$\lambda$	Detachment coefficient	$m^{-1} \ d^{-1}$	10	(c)
*V*	Reactor volume	$m^{3}$	400	(c)
*Q*	Volumetric flow rate	$m^{3} \ d^{-1}$	600	(c)
$N_G$	Number of granules in the reactor	$-$ $-$	$2.4\cdot 10^{10}$	(c)

(a) Batstone et al. (2002; b) Stewart (2003; c) Assumed

## Numerical Simulations and Results

The model has been integrated numerically by developing an original code in MATLAB platform. Hyperbolic PDEs (Eq. (12)) have been integrated by using the method of characteristics, applied for the first time in the biofilm context by D’Acunto and Frunzo (2011), while the method of lines has been adopted for the diffusion-reaction PDEs (Eqs. (15) and (16)). The ordinary differential equations for $\psi ^*_i$ and $S^*_j$ (Eqs. (28) and (29)) have been integrated by using the MATLAB routine ode45, based on a Runge–Kutta method. The time to compute the values of the unknown variables is in the order of hours and depends on the specific target simulation time.

Numerical simulations are performed to describe the formation and evolution of anaerobic granular biofilms, to study the ecological succession occurring in the granule and explore the effects of the main factors on the process. In particular, five numerical studies are carried out: the first numerical study (NS1) describes the de novo granulation process in a bioreactor fed with an influent wastewater rich in sugar; the second study (NS2) investigates the effect of influent composition on granule evolution and ecology; the third study (NS3) explores the role of the attachment phenomenon on granule evolution; the fourth study (NS4) investigates the effects of the biomass density on the transport of soluble substrates and, consequently, on the growth and stratification of biomass within the granule; and the fifth study (NS5) simulates the effects of different detachment regimes on granule size and dynamics. Lastly, a sixth study (NS6) analyses the effects of the number of granules on the process. The values used for the parameters under study are presented in Table 3 for all numerical studies.

The initial concentration of the soluble substrates in the bulk liquid $S^*_{j,0}$ is assumed the same as the influent wastewater. No microbial biomass is present in the influent flow ($\psi ^{in}_i=0$), while non-null initial concentrations of planktonic biomasses in the bulk liquid $\psi ^*_{i,0}$ are set to simulate the reactor inoculated with an anaerobic sludge. In particular, it is considered that the granular reactor is inoculated with the sludge coming from a suspended-based anaerobic reactor and fed with the same influent wastewater. Therefore, the initial concentrations of planktonic species in the bulk liquid (representative of the inoculum) are derived from numerical results of an ADM1-based model (Batstone et al. 2002).

**Table 3:** Initial and boundary conditions and operating parameters adopted in numerical studies

Parameter	NS1	NS2	NS3	NS4	NS5	NS6
	RUN1	RUN2 - RUN4	RUN5 - RUN13	RUN14 - RUN17	RUN18 - RUN25	RUN26 - RUN30
$S^{in}_{Su}$ $[gCOD \ \mathrm {m}^{-3}]$	3500	$varied^1$	3500	3500	3500	3500
$S^{in}_{Bu}$ $[gCOD \ \mathrm {m}^{-3}]$	0	$varied^1$	0	0	0	0
$S^{in}_{Pro}$ $[gCOD \ \mathrm {m}^{-3}]$	0	$varied^1$	0	0	0	0
$S^{in}_{Ac}$ $[gCOD \ \mathrm {m}^{-3}]$	0	$varied^1$	0	0	0	0
$S^{in}_{CH_4}$ $[gCOD \ \mathrm {m}^{-3}]$	0	$varied^1$	0	0	0	0
$\psi ^{*}_{Su,0}$ $[gCOD \ \mathrm {m}^{-3}]$	300	$varied^1$	300	300	300	300
$\psi ^{*}_{Bu,0}$ $[gCOD \ \mathrm {m}^{-3}]$	50	$varied^1$	50	50	50	50
$\psi ^{*}_{Pro,0}$ $[gCOD \ \mathrm {m}^{-3}]$	50	$varied^1$	50	50	50	50
$\psi ^{*}_{Ac,0}$ $[gCOD \ \mathrm {m}^{-3}]$	100	$varied^1$	100	100	100	100
$v_{a,Su}$ $[m \ d^{-1}]$	$3\cdot 10^{-3}$	$3\cdot 10^{-3}$	$varied^1$	$3\cdot 10^{-3}$	$3\cdot 10^{-3}$	$3\cdot 10^{-3}$
$v_{a,Bu}$ $[m \ d^{-1}]$	$3\cdot 10^{-3}$	$3\cdot 10^{-3}$	$varied^1$	$3\cdot 10^{-3}$	$3\cdot 10^{-3}$	$3\cdot 10^{-3}$
$v_{a,Pro}$ $[m \ d^{-1}]$	$3\cdot 10^{-3}$	$3\cdot 10^{-3}$	$varied^1$	$3\cdot 10^{-3}$	$3\cdot 10^{-3}$	$3\cdot 10^{-3}$
$v_{a,Ac}$ $[m \ d^{-1}]$	$150\cdot 10^{-3}$	$150\cdot 10^{-3}$	$varied^1$	$150\cdot 10^{-3}$	$150\cdot 10^{-3}$	$150\cdot 10^{-3}$
$\rho$ $[gCOD \ \mathrm {m}^{-3}]$	120000	120000	120000	$varied^1$	120000	120000
$\lambda$ $[m^{-1} \ d^{-1}]$	10	10	10	10	$varied^1$	10
$N_G$ [$-$ $-$]	$2.4\cdot 10^{10}$	$2.4\cdot 10^{10}$	$2.4\cdot 10^{10}$	$2.4\cdot 10^{10}$	$2.4\cdot 10^{10}$	$varied^1$
*T* [*d*]	300	300	300	300	300	300

$^1$The values used are reported in the text

In granular bioreactors, intense hydrodynamic conditions will improve the aggregation of planktonic cells and the formation of granules. Consequently, for all the simulations reported in this work, the bioreactor volume *V* and the influent flow rate *Q* are assumed constant and equal to $400~\mathrm{m}^3$ and $600~\mathrm{m}^3\, d^{-1}$, respectively, leading to high hydrodynamic velocities and a very low hydraulic retention time (HRT = $0.667 \ d$). Such values are within the range of hydraulic retention times (HRT) values typical of granular biofilm systems (Lim and Kim 2014). Moreover, the organic loading rate (OLR), defined as the amount of daily organic matter treated per unit reactor volume, is set equal to $5.25~\mathrm{kg\, m}^{-3}\, \mathrm{d}^{-1}$. The number of granules $N_G$ has been selected through an iterative procedure which involved the detachment coefficient $\lambda$, with the aim to guarantee a $25\%$ filling ratio (Volcke et al. 2010; Odriozola et al. 2016) by considering $1\,mm$ as steady-state particle radius, an average size representative of these anaerobic granular communities (Trego et al. 2020b; Batstone et al. 2004; Feldman et al. 2017).

Diffusivity of soluble substrates in biofilm is assumed to be $80\%$ of diffusivity in water (Wanner and Gujer 1986). The diffusion coefficients in water for all soluble substrates are taken from Stewart (2003), see Table 2.

The simulation time *T* is fixed to $300 \ d$ for all simulations. This time interval guarantees to achieve the steady-state configuration for all model variables: concentration of soluble substrates $S^*_j(t)$ and planktonic biomasses $\psi ^*_i(t)$ in the bulk liquid; granule size *R*(*t*); sessile biomass fractions $f_i(r,t)$, concentration of soluble substrates $S_j(r,t)$ and planktonic species $\psi _i(r,t)$ within the biofilm.

### NS1—Anaerobic Granulation Process

The first numerical study (NS1) describes the de novo granulation process occurring in a granular reactor fed with sugar: $S^{in}_{Su} = 3500~\mathrm{g\,m}^{-3}$, $S^{in}_{Bu} = S^{in}_{Pro} = S^{in}_{Ac} = S^{in}_{CH_4} = 0$ (RUN1). The initial concentration of the planktonic biomasses (reactor inoculum) is derived from an ADM1-based model following the procedure introduced above: $\psi ^*_{Su,0} = 300~\mathrm{g\,m}^{-3}$, $\psi ^*_{Bu,0} = \psi ^*_{Pro,0} = 50~\mathrm{g\,m}^{-3}$, $\psi ^*_{Ac,0} = 100~\mathrm{g\,m}^{-3}$.

**Fig. 2:**
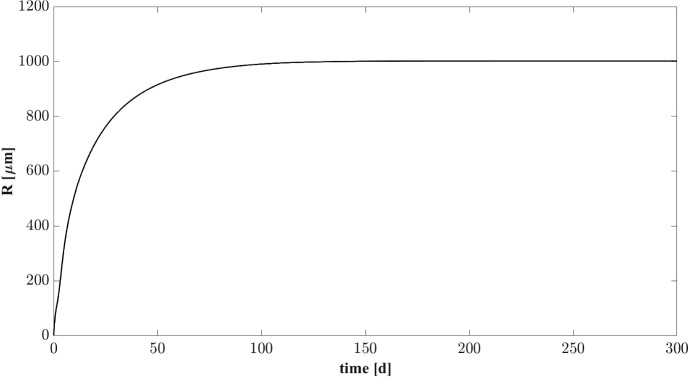
NS1 - Biofilm radius evolution over time. Influent wastewater composition: $S^{in}_{Su} = 3500 \ g~\mathrm{m}^{-3}$ (Sugar), $S^{in}_{Bu} = 0$ (Butyrate), $S^{in}_{Pro} = 0$ (Propionate), $S^{in}_{Ac} = 0$ (Acetate), $S^{in}_{CH_4} = 0$ (Methane)

**Fig. 3:**
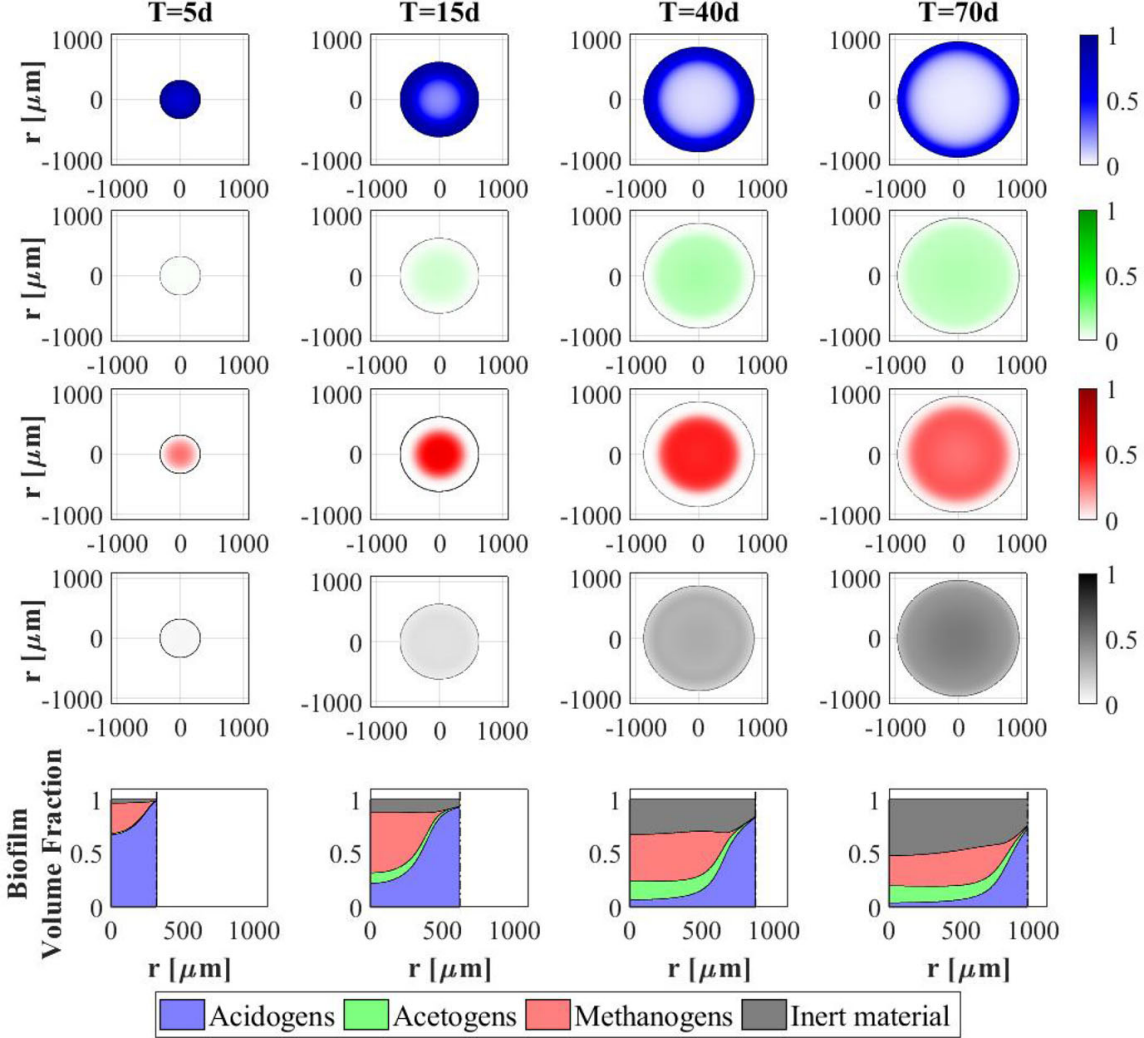
NS1 - Microbial species distribution in the diametrical section and across the radius of the granule, at $T = 5 \ d$, $T = 15 \ d$, $T = 40 \ d$ and $T = 70 \ d$. Influent wastewater composition: $S^{in}_{Su} = 3500 \ g \mathrm{\, m}^{-3}$ (Sugar), $S^{in}_{Bu} = 0$ (Butyrate), $S^{in}_{Pro} = 0$ (Propionate), $S^{in}_{Ac} = 0$ (Acetate), $S^{in}_{CH_4} = 0$ (Methane) (color figure online)

**Fig. 4:**
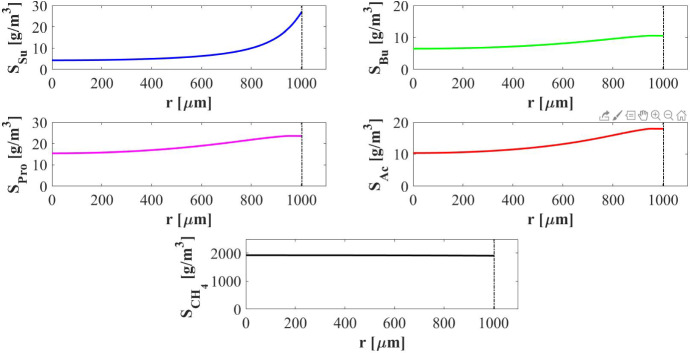
NS1 - Distribution of soluble substrates along the granule radius at $T=300 \ d$. $S_{Su}$: Sugar, $S_{Bu}$: Butyrate, $S_{Pro}$: Propionate, $S_{Ac}$: Acetate, $S_{CH_4}$: Methane. Influent wastewater composition: $S^{in}_{Su} = 3500 \ g~\mathrm{m}^{-3}$ (Sugar), $S^{in}_{Bu} = 0$ (Butyrate), $S^{in}_{Pro} = 0$ (Propionate), $S^{in}_{Ac} = 0$ (Acetate), $S^{in}_{CH_4} = 0$ (Methane) (color figure online)

**Fig. 5:**
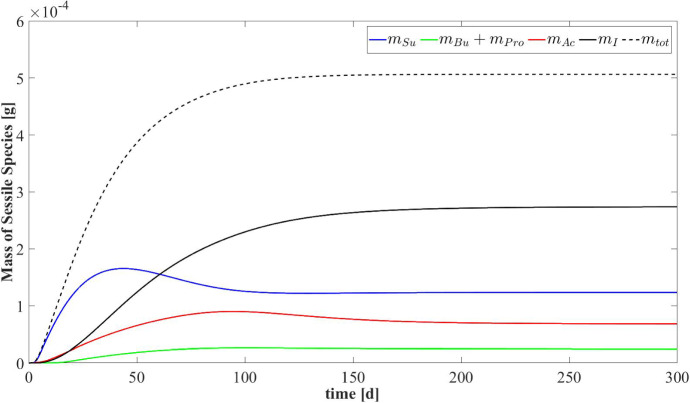
NS1 - Evolution of mass of sessile species within the reactor. $m_{Su}$: mass of sugar fermenters, $m_{Bu}$: mass of butyrate consumers, $m_{Pro}$: mass of propionate consumers, $m_{Ac}$: mass of acetoclastic methanogens, $m_{tot}$: total sessile mass. Influent wastewater composition: $S^{in}_{Su} = 3500 \ g~\mathrm{m}^{-3}$ (Sugar), $S^{in}_{Bu} = 0$ (Butyrate), $S^{in}_{Pro} = 0$ (Propionate), $S^{in}_{Ac} = 0$ (Acetate), $S^{in}_{CH_4} = 0$ (Methane) (color figure online)

**Fig. 6:**
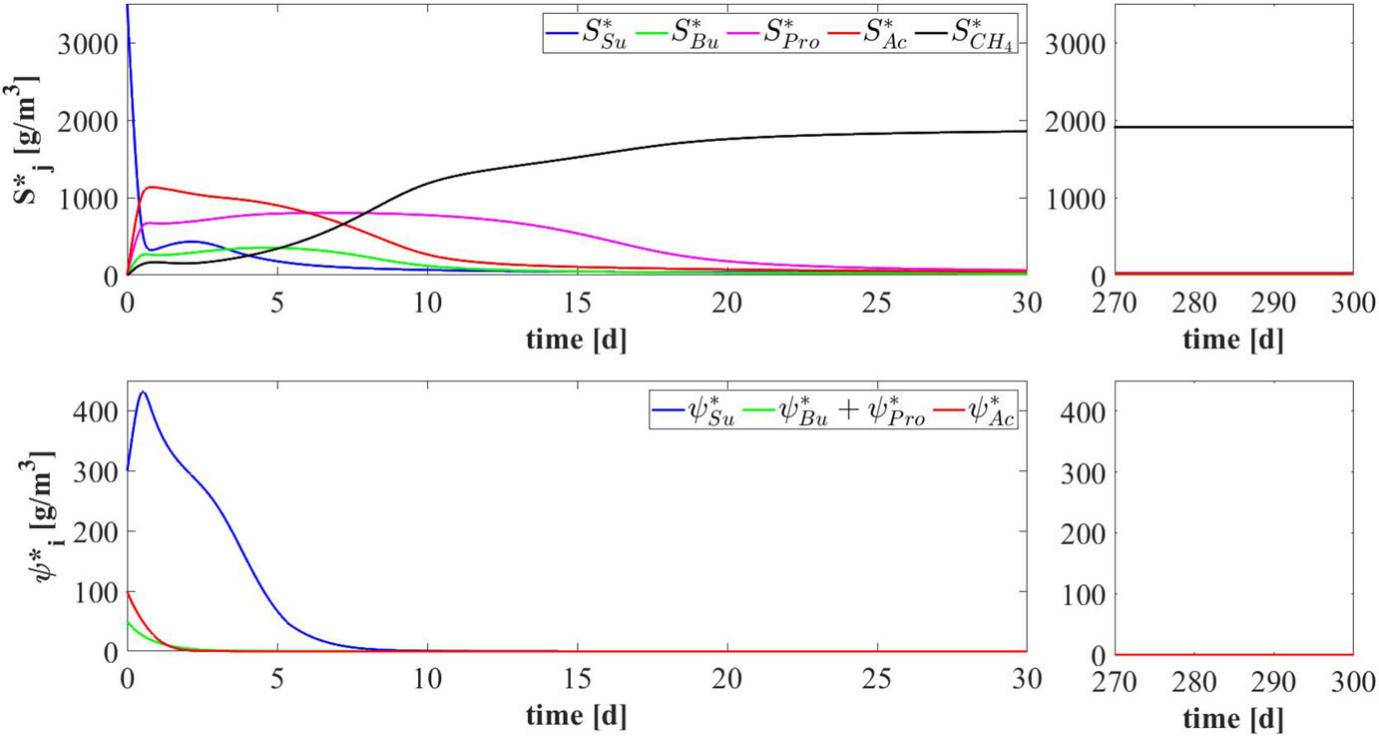
NS1 - Evolution of soluble substrates (top) and planktonic biomass (bottom) concentrations within the bulk liquid. $S^*_{Su}$: Sugar, $S^*_{Bu}$: Butyrate, $S^*_{Pro}$: Propionate, $S^*_{Ac}$: Acetate, $S^*_{CH_4}$: Methane, $\psi ^*_{Su}$: Sugar fermenters, $\psi ^*_{Bu}$: Butyrate consumers, $\psi ^*_{Pro}$: Propionate consumers, $\psi ^*_{Ac}$: Acetoclastic methanogens. Influent wastewater composition: $S^{in}_{Su} = 3500 \ g~\mathrm{m}^{-3}$ (Sugar), $S^{in}_{Bu} = 0$ (Butyrate), $S^{in}_{Pro} = 0$ (Propionate), $S^{in}_{Ac} = 0$ (Acetate), $S^{in}_{CH_4} = 0$ (Methane) (color figure online)

Numerical results are summarized in Figs. 2–6. In Fig. 2 the evolution of the granule radius *R*(*t*) over time is reported. A vanishing initial value is assigned to *R*(*t*) at $t=0$ ($R(0) = 0$). During the first days, the granulation process has its maximum intensity and the granule size increases. The variation of *R*(*t*) is almost exhausted during the first 70–100 days, after which it reaches a steady-state value of about $1~\mathrm{mm}$.

In Fig. 3 the distribution of sessile species within the granule is shown at different times. After $5 \ d$ the granule has a radius of about $0.3~\mathrm{mm}$ and is constituted mostly by acidogens (blue) which are favoured by the high concentration of sugar initially present in the bulk liquid. However, the granule core is also composed of methanogens (red) which have high propensity to attach due to their filamentous structures and aggregation properties. At $T=15 \ d$, the consumption of sugar and the production of volatile fatty acids (VFAs) in large amount by acidogenesis affects the biomass distribution: the methanogenic core grows while the acidogens occupy the outer layer of the granule, and the acetogens (green) and inert (black) fractions become be visible. For later times (40–70 $\mathrm{d}$), the radius almost reaches the steady-state value, a significant amount of inert material accumulates (especially in the innermost part of the domain), and homogeneous fractions of methanogens and acetogens are found throughout the granule-except the outermost part, where a thin layer of acidogens is established. This stratification is due to the distribution of soluble substrate concentrations along the radius of the granule, shown at $T=300~\mathrm{d}$ in Fig. 4. The concentration of sugar in the outermost layers promotes the growth of acidogens, which have a higher maximum growth rate than the other species. Otherwise, the substrate concentrations reduce towards the centre of the granule. Shortage of substrates affects acidogens more than other species, due to their high sugar half saturation constant (see Table 2). Then, since the acetate half saturation constant for methanogens is very low, they are able to grow even under low substrate concentrations and prevail in this central area.

Figure 5 presents the trend of each microbial species within the granule over time. This result confirms the microbial succession described above. The biofilm is initially constituted predominantly by acidogens $m_{Su}$ (blue) and methanogens $m_{Ac}$ (red). Their mass within the biofilm achieves a maximum and then decreases to a steady-state value when the substrates required by their metabolism (sugar and acetate, respectively) are limited and the decay process prevails. The growth process of acetogens $m_{Bu}+m_{Pro}$ and the accumulation of inert $m_{I}$ are slower and take place over a longer period. However, all microbial species exhibit steady-state values 150–170 days after granule genesis. Furthermore, the total microbial mass $m_{tot}$ (dashed black line) follows the trend of the radius reported in Fig. 2. Indeed, assuming a constant density $\rho$, the variation of mass within the granule is related to the variation of volume.

Lastly, the trends of soluble substrates and planktonic biomass within the bulk liquid are shown in Fig. 6. In the initial phase, the biofilm granules are small, and the consumption and production of soluble substrates are governed by planktonic biomass. In particular, planktonic sugar fermenters $\psi ^*_{Su}$ (blue in Fig. 6-bottom) degrade sugar $S^*_{Su}$ (blue in Fig. 6-top) and produce volatile fatty acids (VFAs): i.e. butyrate $S^*_{Bu}$ (green in Fig. 6-top), propionate $S^*_{Pro}$ (magenta in Fig. 6-top) and acetate $S^*_{Ac}$ (red in Fig. 6-top). Meanwhile, the concentration of all planktonic biomass within the bulk liquid is reduced due to two distinct phenomena: part is converted in sessile biomass during the granulation process and part is rapidly washed out due to the hydrodynamic conditions (i.e. low HRTs in ‘high rate’ bioreactors). For these reasons, no microbial species in planktonic form is present within the reactor after 5–7 days. After the washout of the planktonic biomass, the substrates trend is influenced exclusively by the sessile metabolic activity: the residual sugar $S^*_{Su}$ and VFAs ($S^*_{Bu}$, $S^*_{Pro}$ and $S^*_{Ac}$) are consumed with different velocities according to the consumption rate of the corresponding sessile microbial species and significant amount of methane $S^*_{CH_4}$ (black in Fig. 6-top) is produced. After 30 days, substrates concentrations within the bulk liquid reach a steady-state value. High concentrations of methane (the end product of the AD process), and negligible concentrations of sugar and VFAs, are found in the bioreactor and in the effluent.

### NS2—Effects of Influent Wastewater Composition

**Fig. 7:**
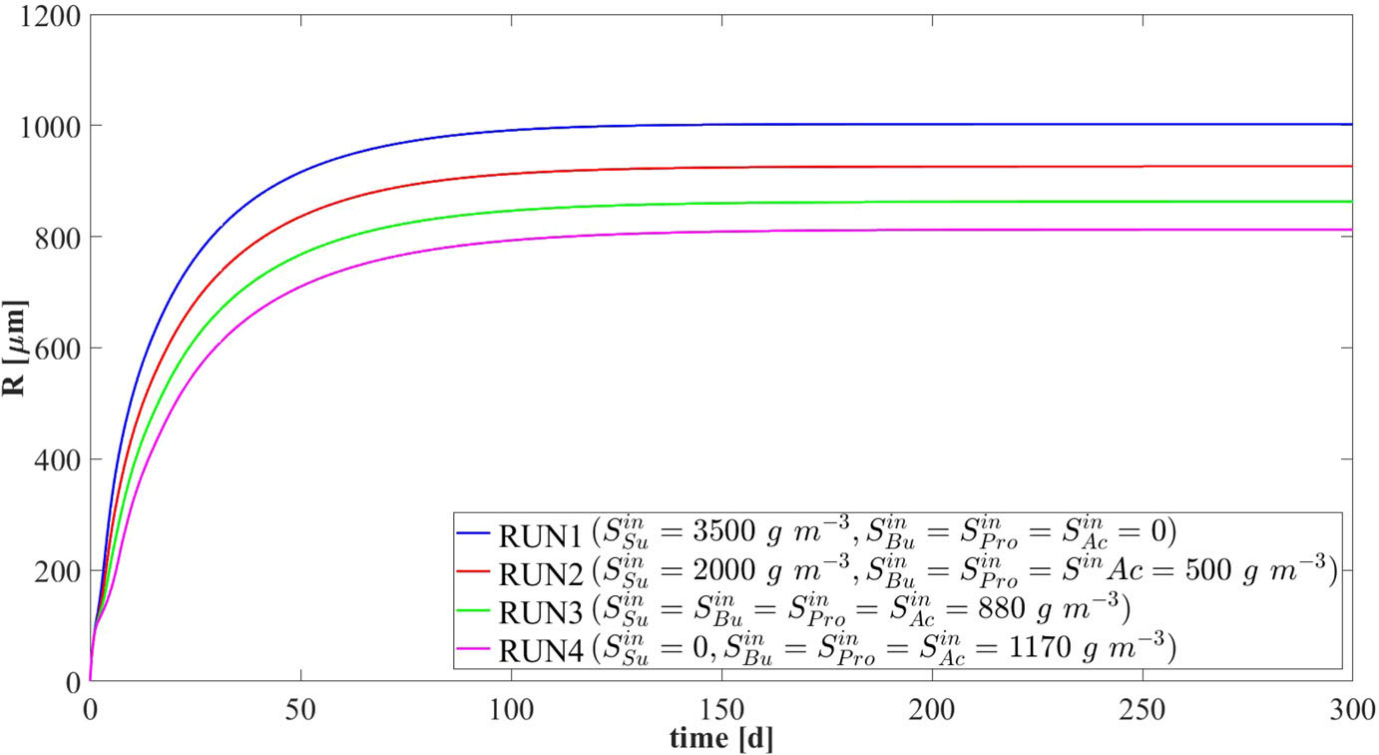
NS2 - Biofilm radius evolution over time for different influent wastewater compositions. $S^{in}_{Su}$: Sugar, $S^{in}_{Bu}$: Butyrate, $S^{in}_{Pro}$: Propionate, $S^{in}_{Ac}$: Acetate, $S^{in}_{CH_4}$: Methane (color figure online)

**Fig. 8:**
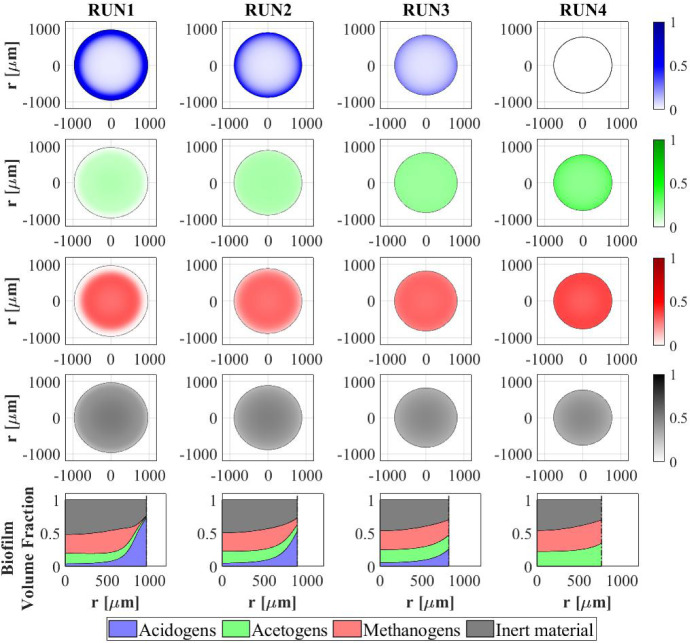
NS2 - Microbial species distribution in the diametrical section and across the radius of the granule at $T\,{=}\,70 \ d$, for different influent wastewater compositions. RUN1: $S^{in}_{Su}\,{=}\,3500 \ g \ m^{-3}$, $S^{in}_{Bu}\,{=}\,S^{in}_{Pro}\,{=}\,S^{in}_{Ac}\,{=}\,S^{in}_{CH_4}\,{=}\, 0$; RUN2: $S^{in}_{Su}\,{=}\,2000 \ g~\mathrm{m}^{-3}$, $S^{in}_{Bu}\,{=}\, S^{in}_{Pro}\,{=}\,S^{in}_{Ac}\,{=}\,500 \ g~\mathrm{m}^{-3}$, $S^{in}_{CH_4}\,{=}\,0$; RUN3: $S^{in}_{Su}\,{=}\,S^{in}_{Bu}\,{=}\,S^{in}_{Pro}\,{=}\,S^{in}_{Ac}\,{=}\,880 \ g~\mathrm{m}^{-3}$, $S^{in}_{CH_4}\,{=}\,0$; RUN4: $S^{in}_{Su}\,{=}\,0$, $S^{in}_{Bu}\,{=}\,S^{in}_{Pro}\,{=}\,S^{in}_{Ac}\,{=}\,1170 \ g~\mathrm{m}^{-3}$, $S^{in}_{CH_4}\,{=}\,0$. $S^{in}_{Su}$: Sugar, $S^{in}_{Bu}$: Butyrate, $S^{in}_{Pro}$: Propionate, $S^{in}_{Ac}$: Acetate, $S^{in}_{CH_4}$: Methane (color figure online)

**Fig. 9:**
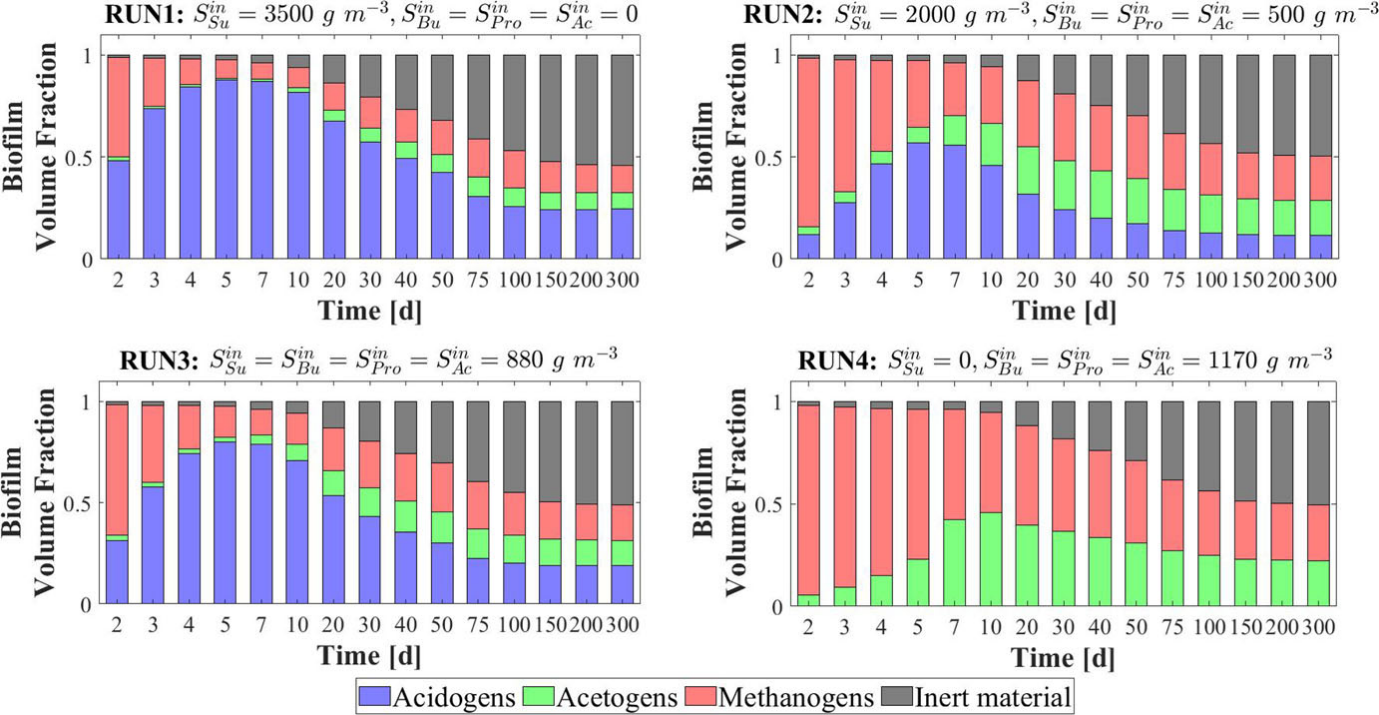
NS2 - Relative abundances of microbial species within the granule at several times under different influent wastewater compositions. $S^{in}_{Su}$: Sugar, $S^{in}_{Bu}$: Butyrate, $S^{in}_{Pro}$: Propionate, $S^{in}_{Ac}$: Acetate, $S^{in}_{CH_4}$: Methane (color figure online)

**Fig. 10:**
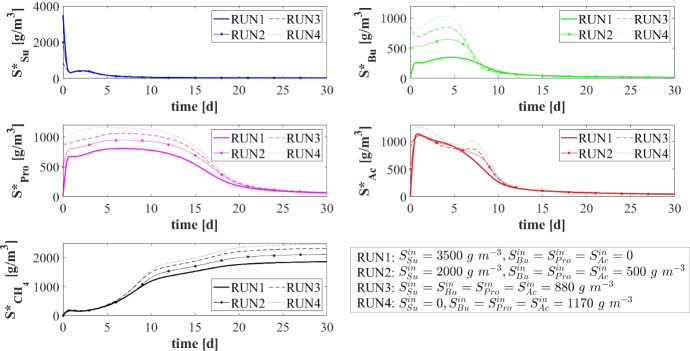
NS2 - Evolution of soluble substrates concentrations within the bulk liquid for different influent wastewater compositions. $S^*_{Su}$: Sugar, $S^*_{Bu}$: Butyrate, $S^*_{Pro}$: Propionate, $S^*_{Ac}$: Acetate, $S^*_{CH_4}$: Methane (color figure online)

**Fig. 11:**
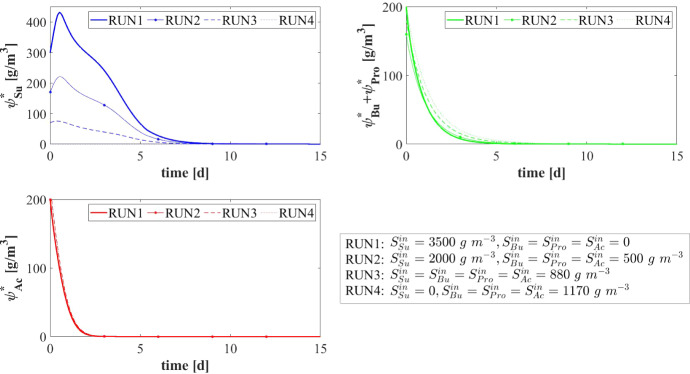
NS2 - Evolution of planktonic biomass concentrations within the bulk liquid for different influent wastewater compositions. $\psi ^*_{Su}$: Sugar fermenters, $\psi ^*_{Bu}$: Butyrate consumers, $\psi ^*_{Pro}$: Propionate consumers, $\psi ^*_{Ac}$: Acetoclastic methanogens (color figure online)

The results presented in the previous section describe the dynamic evolution and the steady-state configuration of anaerobic granular biofilms growing in a sugar-fed bioreactor. However, the composition of the influent wastewater affects the granulation process and regulates the ecological succession and the growth of individual species. Since the influent wastewater treated in anaerobic granular systems originates from any one of various applications and sources, and thus presents variable compositions of the organic load, it is interesting to compare the model results for different types of influent wastewater. In particular, in this study (NS2) different influent compositions and bioreactor inocula are set as model input: (RUN2: $S^{in}_{Su}\,{=}\,2000\, \mathrm{g\,m}^{-3}$, $S^{in}_{Bu}\,{=}\,S^{in}_{Pro}\,{=}\,S^{in}_{Ac}\,{=}\,500\, \mathrm{g\,m}^{-3}$, $S^{in}_{CH_4}\,{=}\,0$, $\psi ^*_{Su,0}\,{=}\,170\, \mathrm{g\,m}^{-3}$, $\psi ^*_{Bu,0} \,{=}\,\psi ^*_{Pro,0} \,{=}\,40\, \mathrm{g\,m}^{-3}$, $\psi ^*_{Ac,0} \,{=}\,100\, \mathrm{g\,m}^{-3}$; RUN3: $S^{in}_{Su}\,{=}\,S^{in}_{Bu}\,{=}\,S^{in}_{Pro}\,{=}\,S^{in}_{Ac}\,{=} 880 \, \mathrm{g\,m}^{-3}$, $S^{in}_{CH_4}\,{=}\,0$, $\psi ^*_{Su,0}{=}\,70\, \mathrm{g\,m}^{-3}$, $\psi ^*_{Bu,0}{=}\,50\, \mathrm{g\,m}^{-3}, \psi ^*_{Pro,0}\,{=}\,40\, \mathrm{g\,m}^{-3}$, $\psi ^*_{Ac,0}\,{=}110\, \mathrm{g\,m}^{-3}$; RUN4: $S^{in}_{Su}{=}\,S^{in}_{CH_4}{=}\,0$, $S^{in}_{Bu}{=}\,S^{in}_{Pro}\,{=}\,S^{in}_{Ac}\,{=}1170\, \mathrm{g\,m}^{-3}$, $\psi ^*_{Su,0}\,{=}\,0$, $\psi ^*_{Bu,0}\,{=}\,60\, \mathrm{g\,m}^{-3}, \psi ^*_{Pro,0}\,{=}\,40\, \mathrm{g\,m}^{-3}$, $\psi ^*_{Ac,0}\,{=}\,110\, \mathrm{g\,m}^{-3}$). These cases have been compared with the case of reactor fed with only sugar (RUN1). The results are summarized in Figs. 7–11.

Figure 7 shows the trend of the granule radius *R*(*t*) over time. Granules of different sizes are formed. These differences are related to the sessile biomass growth, which varies according to the substrates present in the influent wastewater. Specifically, sessile growth is affected by anabolic and catabolic pathways of the microbial metabolism: the yield of acidogens on sugar $Y_{Su}$ is higher than the yields of the other species, and thus, the amount of acidogenic biomass grown per unit of substrate consumed is higher than the other species. Therefore, the steady-state size of the granule increases with increasing sugar concentration in the influent $S^{in}_{Su}$. For $S^{in}_{Su}=0$ (RUN4), the granule achieves the smallest size.

The distribution of sessile biomass within the granule in the four cases is reported in Fig. 8, at $T = 70 \ d$. As the sugar concentration in the influent $S^{in}_{Su}$ increases, the fraction of acidogens $f_{Su}$ (blue) increases, especially in the outer part of the granule, where there is maximum availability of substrate. When the sugar concentration $S^{in}_{Su}$ decreases from $3500~\mathrm{g\,m}^{-3}$ (RUN1) to $880~\mathrm{g\,m}^{-3}$ (RUN3), the acidogenic fraction present in the external part of the biofilm significantly reduces. Obviously, no acidogens are found within the granule when sugar is absent in the influent wastewater (RUN4). In addition, as the concentration of butyrate $S^{in}_{Bu}$, propionate $S^{in}_{Pro}$ and acetate $S^{in}_{Ac}$ in the influent increases going from RUN1 to RUN4, an increase in the fractions of acetogens $f_{Bu}+f_{Pro}$ (green) and methanogens $f_{Ac}$ (red) is observed.

The relative abundance of sessile microbial species is reported for different simulation times in Fig. 9. When sugar is present in the influent (RUN1, RUN2, RUN3), the initial phase of the granulation is governed by acidogens (which have a higher growth rate) and methanogens (which have high attachment velocities). The acidogenic fraction (blue) reaches a maximum after $7\ d$ and then decreases when the availability of sugar in the bulk liquid reduces. When sugar is not present in the influent (RUN4), the granulation process is dominated by methanogens (red) and acetogens in small amounts (green). In all four cases, the maximum fraction of methanogens is observed at the beginning of the process due to their granulation properties. Then, the methanogenic fraction reduces due to the decay process and the competition with acidogens and acetogens. Furthermore, the acetogenic fraction is negligible in all cases during the initial phase of the granulation and grows when other microbial species become less competitive, and sugar is converted to butyrate and propionate. The microbial relative abundances related to the steady-state value confirm the results introduced in Fig. 8: the fraction of acidogens increases with the increase of the sugar in the influent; the fractions of methanogens and acetogens increase with increasing VFAs in the influent; in all cases, inactive biomass (black) represents approximately 50% of the total sessile biomass within the granule.

The model results related to the bulk liquid are summarized in Figs. 10 and 11, which show how the concentration of soluble substrates and planktonic biomass changes over time. As reported in Fig. 10, the composition of the influent wastewater affects the trend of the substrates mostly in the initial phase. In all cases, the AD process is completed in about 30 days: sugar (blue), butyrate (green), propionate (magenta) and acetate (red) are totally consumed and the concentration of methane (black) achieves a steady-state value. Notably, different productions of methane are observed as the composition of the influent changes. Concerning the concentration of planktonic biomass shown in Fig. 11, the concentration of acidogens (blue) is affected by the composition of the influent in the initial phase of the granulation process (0-7 days). Conversely, the concentration of acetogens (green) and methanogens (red) in planktonic form have low growth rates and are washed out due to the dilution phenomenon even when significant concentrations of VFAs are present in the influent.

**Fig. 12:**
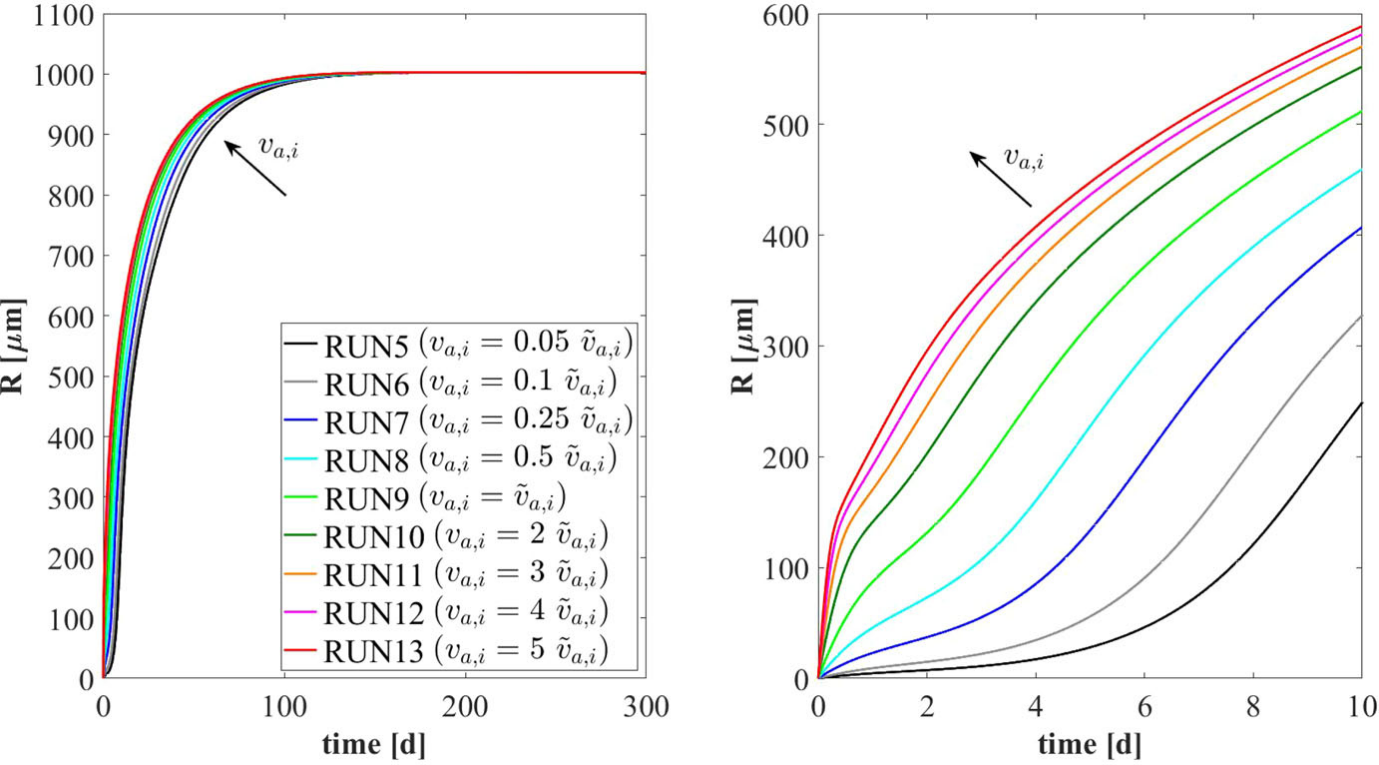
NS3 - Biofilm radius evolution over time for different attachment velocities $v_{a,i}$ (left), with a focus on the first 10 days (right). ${\tilde{v}}_{a,i}$: value of attachment velocity of the *i*th planktonic species set in RUN1. Influent wastewater composition: $S^{in}_{Su} = 3500 \ g~\mathrm{m}^{-3}$ (Sugar), $S^{in}_{Bu} = 0$ (Butyrate), $S^{in}_{Pro} = 0$ (Propionate), $S^{in}_{Ac} = 0$ (Acetate), $S^{in}_{CH_4} = 0$ (Methane) (color figure online)

### NS3—Effects of Granulation Properties

The characteristics of the microbial community play a fundamental role in the anaerobic granulation process. In particular, it is affected by the granulating properties of the planktonic biomass present in the bioreactor. In the management of full-scale bioreactors, several strategies are pursued to improve the aggregation properties of the microbial community, reduce the time required for granulation and improve the efficiency of the wastewater treatment process. For example, the direct addition of the quorum sensing molecule acyl homoserine lactone (AHL) during granule formation might remarkably improve the granulation process in granular bioreactors (Li et al. 2015; Zhang et al. 2012; De Vrieze and Verstraete 2016). Equally, bioaugmentation is regarded as a promising method to improve granulation and reduce the start-up in full-scale plants (Guiot et al. 2000; Nancharaiah et al. 2008; Jin et al. 2014). The addition to bioreactors of selected microbial cultures depth in self-aggregation has been described (Bathe et al. 2005). Overall, such strategies positively alter the granulation properties of planktonic microbial communities.

**Fig. 13:**
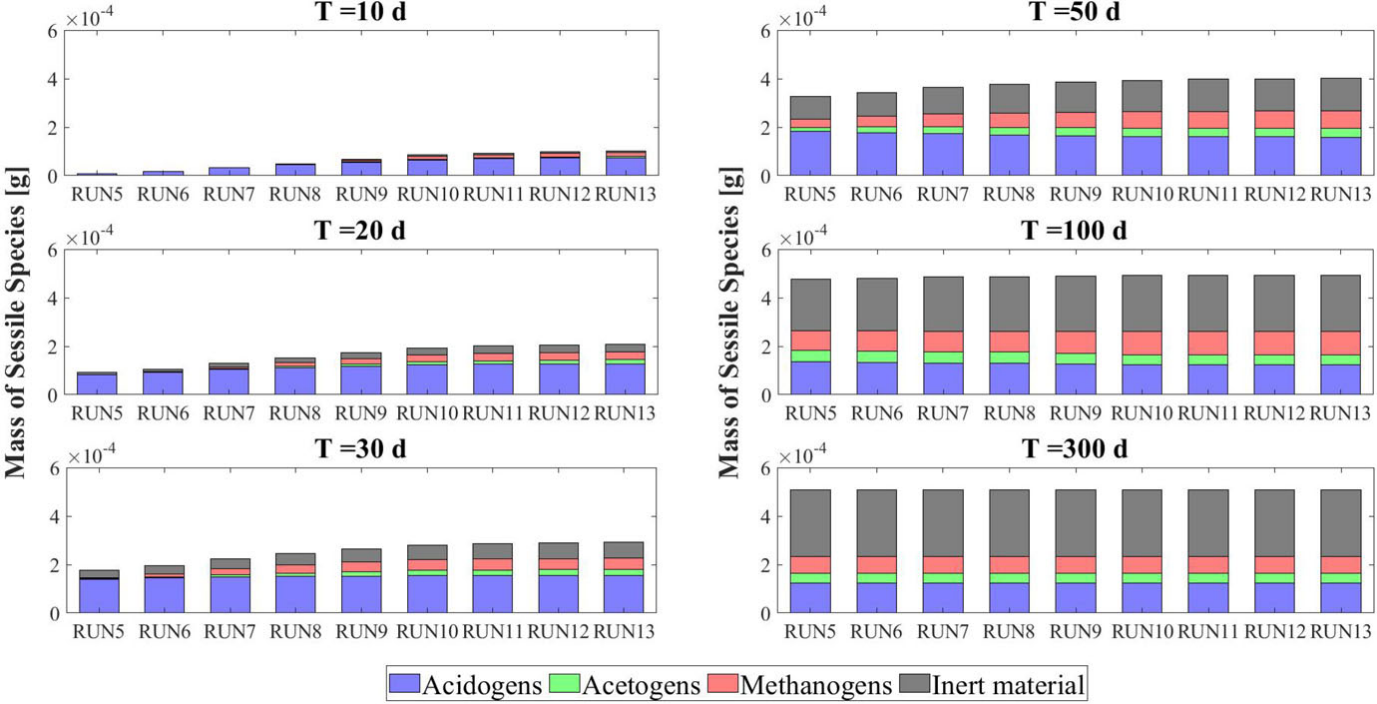
NS3 - Mass of microbial species within the granule at $T = 10 \ d$, $T = 20 \ d$, $T = 30 \ d$, $T = 50 \ d$, $T = 100 \ d$ and $T = 300 \ d$ under different attachment velocities $v_{a,i}$. RUN5: $v_{a,i} = 0.05 \ {\tilde{v}}_{a,i}$, RUN6: $v_{a,i} = 0.1 \ {\tilde{v}}_{a,i}$, RUN7: $v_{a,i} = 0.25 \ {\tilde{v}}_{a,i}$, RUN8: $v_{a,i} = 0.5 \ {\tilde{v}}_{a,i}$, RUN9: $v_{a,i} = \ {\tilde{v}}_{a,i}$, RUN10: $v_{a,i} = 2 \ {\tilde{v}}_{a,i}$, RUN11: $v_{a,i} = 3 \ {\tilde{v}}_{a,i}$, RUN12: $v_{a,i} = 4 \ {\tilde{v}}_{a,i}$, RUN13: $v_{a,i} = 5 \ {\tilde{v}}_{a,i}$. ${\tilde{v}}_{a,i}$: value of attachment velocity of the *i*th planktonic species set in RUN1. Influent wastewater composition: $S^{in}_{Su} = 3500 \ g~\mathrm{m}^{-3}$ (Sugar), $S^{in}_{Bu} = 0$ (Butyrate), $S^{in}_{Pro} = 0$ (Propionate), $S^{in}_{Ac} = 0$ (Acetate), $S^{in}_{CH_4} = 0$ (Methane) (color figure online)

**Fig. 14:**
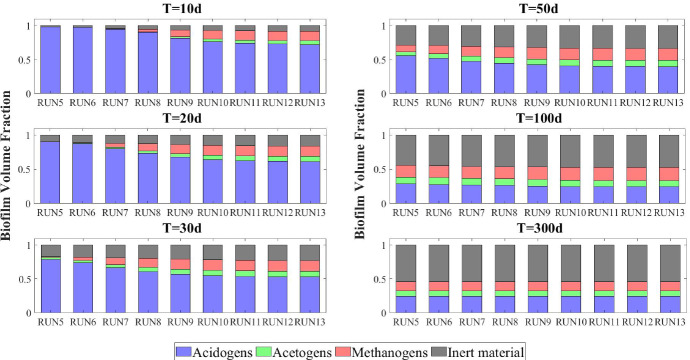
NS3 - Relative abundances of microbial species within the granule at $T = 10 \ d$, $T = 20 \ d$, $T = 30 \ d$, $T = 50 \ d$, $T = 100 \ d$ and $T = 300 \ d$ under different attachment velocities $v_{a,i}$. RUN5: $v_{a,i} = 0.05 \ {\tilde{v}}_{a,i}$, RUN6: $v_{a,i} = 0.1 \ {\tilde{v}}_{a,i}$, RUN7: $v_{a,i} = 0.25 \ {\tilde{v}}_{a,i}$, RUN8: $v_{a,i} = 0.5 \ {\tilde{v}}_{a,i}$, RUN9: $v_{a,i} = \ {\tilde{v}}_{a,i}$, RUN10: $v_{a,i} = 2 \ {\tilde{v}}_{a,i}$, RUN11: $v_{a,i} = 3 \ {\tilde{v}}_{a,i}$, RUN12: $v_{a,i} = 4 \ {\tilde{v}}_{a,i}$, RUN13: $v_{a,i} = 5 \ {\tilde{v}}_{a,i}$. ${\tilde{v}}_{a,i}$: value of attachment velocity of the *i*th planktonic species set in RUN1. Influent wastewater composition: $S^{in}_{Su} = 3500 \ g~\mathrm{m}^{-3}$ (Sugar), $S^{in}_{Bu} = 0$ (Butyrate), $S^{in}_{Pro} = 0$ (Propionate), $S^{in}_{Ac} = 0$ (Acetate), $S^{in}_{CH_4} = 0$ (Methane) (color figure online)

In this framework, a numerical study (NS3) is conducted to investigate the effects on granulation of the granulating properties of the biomass. For this purpose, nine simulations (RUN5 - RUN13) are carried out with different attachment velocities $v_{a,i}$. The nine values of $v_{a,i}$ used are chosen by increasing and reducing the default values (presented in Table 2) through different multiplication factors (0.05, 0.1, 0.25, 0.5, 1, 2, 3, 4, 5). The concentration of soluble substrates in the influent wastewater $S^{in}_j$ and the initial concentration of planktonic biomass in the bioreactor $\psi ^*_{i,0}$ set for this numerical study are the same used in NS1 and are reported in Table 3.

**Fig. 15:**
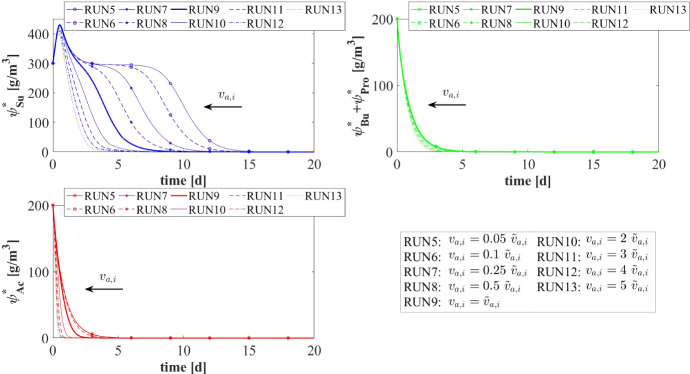
NS3 - Evolution of planktonic biomass concentrations within the bulk liquid for different attachment velocities $v_{a,i}$. $\psi ^*_{Su}$: Sugar fermenters, $\psi ^*_{Bu}$: Butyrate consumers, $\psi ^*_{Pro}$: Propionate consumers, $\psi ^*_{Ac}$: Acetoclastic methanogens. ${\tilde{v}}_{a,i}$: value of attachment velocity of the *i*th planktonic species set in RUN1. Influent wastewater composition: $S^{in}_{Su} = 3500 \ g~\mathrm{m}^{-3}$ (Sugar), $S^{in}_{Bu} = 0$ (Butyrate), $S^{in}_{Pro} = 0$ (Propionate), $S^{in}_{Ac} = 0$ (Acetate), $S^{in}_{CH_4} = 0$ (Methane) (color figure online)

**Fig. 16:**
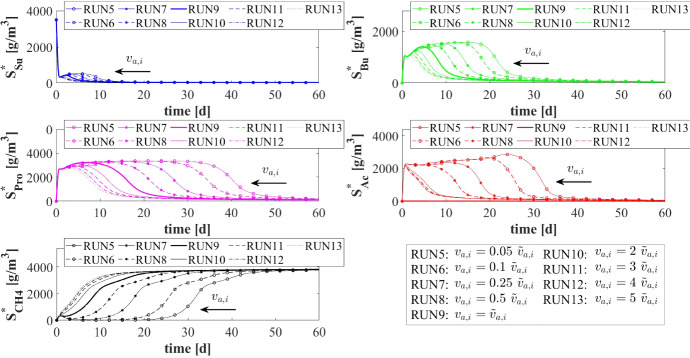
NS3 - Evolution of soluble substrates concentrations within the bulk liquid for different attachment velocities $v_{a,i}$. $S^*_{Su}$: Sugar, $S^*_{Bu}$: Butyrate, $S^*_{Pro}$: Propionate, $S^*_{Ac}$: Acetate, $S^*_{CH_4}$: Methane. ${\tilde{v}}_{a,i}$: value of attachment velocity of the *i*th planktonic species set in RUN1. Influent wastewater composition: $S^{in}_{Su} = 3500 \ g~\mathrm{m}^{-3}$ (Sugar), $S^{in}_{Bu} = 0$ (Butyrate), $S^{in}_{Pro} = 0$ (Propionate), $S^{in}_{Ac} = 0$ (Acetate), $S^{in}_{CH_4} = 0$ (Methane) (color figure online)

The results of this study are reported in Figs. 12-16. The temporal evolution of the granule radius *R*(*t*) is shown in Fig. 12. From Fig. 12 (right) it is clear that different attachment velocities $v_{a,i}$ lead to different growth rates of the granule in the initial phase of the process: when the inoculated microbial community is more inclined to grow in sessile form, the granulation process occurs faster and the granule reaches the steady-state size sooner. However, such steady-state size is not dependent on the attachment velocity. Indeed, the profiles of *R*(*t*) for different $v_{a,i}$ get closer over time and reach the same steady-state value.

Figures 13 and 14 report the mass and the relative abundance of the different sessile microbial species within the granule, respectively. Again, relevant differences concern the initial phase ($$ T = 10$$-$$20 \ d $$), when the total sessile mass, proportional to the granule size, is higher in the case of more intense attachment process. However, after long times both the total sessile mass and the relative abundance of individual microbial species within the granule are no longer affected by $$v_{a,i}$$ and all simulations achieve the same steady-state configuration.

Other interesting results refer to the effects that the granulation process has on the planktonic biomass $\psi ^*_{i}$ (Fig. 15) and soluble substrates $S^*_{j}$ (Fig. 16) within the bioreactor. The concentration profiles of the planktonic acetogens $\psi ^*_{Bu}+\psi ^*_{Pro}$ (green) and methanogens $\psi ^*_{Ac}$ (red) in the bulk liquid, as shown in Fig. 15, are not very sensitive to the variation of $v_{a,i}$. Indeed, the reduction of the planktonic biomass $\psi ^*_{i}$ depends on two phenomena: attachment and dilution. The reduction of $\psi ^*_{Bu}, \psi ^*_{Pro}$ and $\psi ^*_{Ac}$ occurs in the initial phase of the process, when the granules are small and the attachment flux of planktonic biomass (proportional to the granule surface *A*(*t*)) has limited effects on the properties of the bulk liquid. On the other hand, the dilution process is prominent: the hydrodynamic conditions (i.e. high flow rate, low HRT) and slow metabolic growth (due to low maximum growth rates and limited substrate) lead to washout of planktonic acetogens and methanogens. This dilution process is not affected by granulation properties and therefore leads to similar profiles by varying $v_{a,i}$. Conversely, the planktonic acidogens $\psi ^*_{Su}$ (blue) have higher growth rates and optimal conditions to grow (sugar-rich influent). Hence, they are retained in the bioreactor for longer and are depopulated mainly due to the granulation process, which is strongly influenced by $v_{a,i}$. Consequently, different values of $v_{a,i}$ correspond to different profiles of planktonic acidogens $\psi ^*_{Su}$: the higher $v_{a,i}$, the faster the reduction of the concentration $\psi ^*_{Su}$ in the bulk liquid.

Figure 16 shows the trend of soluble substrates within the bioreactor. As just mentioned, in the first few days the granules have a small size and the consumption and production of soluble substrates mainly depend on the metabolic activity of the planktonic biomass. Consequently, in the initial phase the trends of soluble substrates are not affected by attachment conditions. For later times, the granule size and the amount of sessile biomass in the bioreactor increase, and the trend of the substrates becomes more sensitive to $v_{a,i}$: for small values of $v_{a,i}$ the concentrations of soluble substrates reach steady-state conditions in 40-50 days, while for high values of $v_{a,i}$ half the time is required to reach a steady-state configuration.

### NS4—Effects of Biomass Density

Biomass density of the granules involved in granular biofilm systems is highly variable due to several factors, such as hydrodynamic conditions, shear forces and production. Firstly, high shear forces lead to stronger and denser granules, while weaker and more porous granule structures develop under lower shear forces (Liu and Tay 2002; Di Iaconi et al. 2006; Tay et al. 2001). Moreover, EPS production is generally thought to increase cell surface hydrophobicity and promote the formation of a sticky matrix favouring granulation of new cells or flocs (Trego et al. 2020b). Thus, EPS positively influences the granulation process, contributing to the maintenance of the structural integrity of the biofilm matrix and improved biomass density. Biomass density is a crucial property of granular biofilms because it regulates the mass transfer, the consumption of soluble substrates within the granules and, consequently, the growth of microbial cells and the dynamics of the granules. As a result, granules of different densities typically have different sizes and are characterized by different microbial stratifications.

**Fig. 17:**
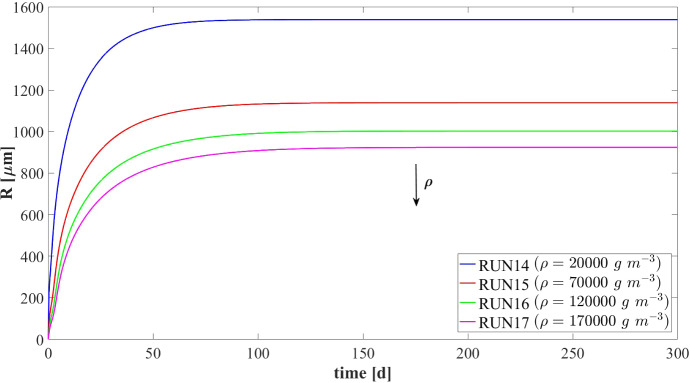
NS4 - Biofilm radius evolution over time for different biomass densities $\rho$. Influent wastewater composition: $S^{in}_{Su} = 3500 \ g~\mathrm{m}^{-3}$ (Sugar), $S^{in}_{Bu} = 0$ (Butyrate), $S^{in}_{Pro} = 0$ (Propionate), $S^{in}_{Ac} = 0$ (Acetate), $S^{in}_{CH_4} = 0$ (Methane) (color figure online)

**Fig. 18:**
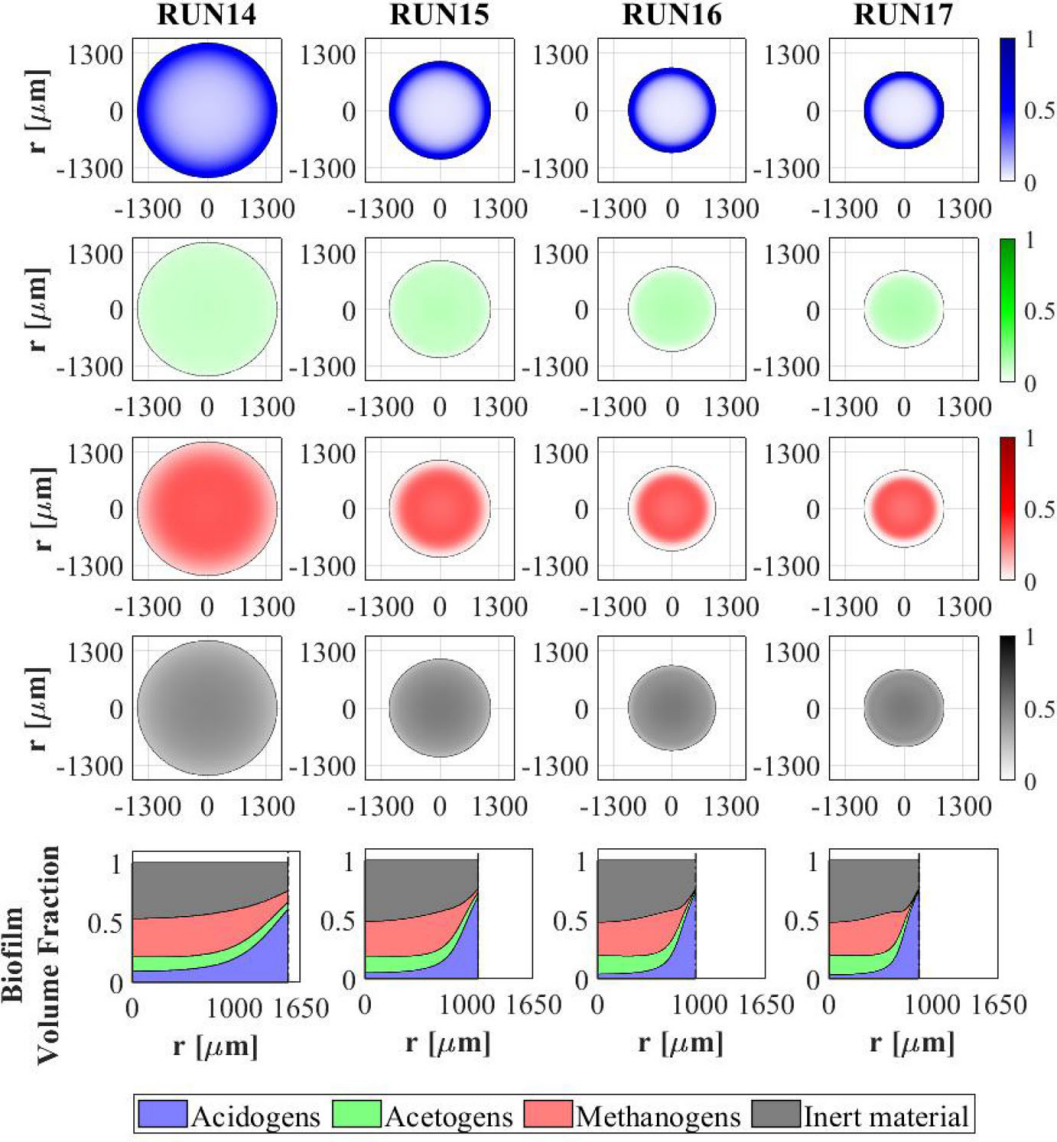
NS4 - Microbial species distribution in the diametrical section and across the radius of the granule at $T = 70 \ d$, for different biomass densities. RUN14: $\rho = 20000 \ g~\mathrm{m}^{-3}$, RUN15: $\rho = 70000 \ g~\mathrm{m}^{-3}$, RUN16: $\rho = 120000 \ g~\mathrm{m}^{-3}$, RUN17: $\rho = 170000 \ g~\mathrm{m}^{-3}$. Influent wastewater composition: $S^{in}_{Su} = 3500 \ g~\mathrm{m}^{-3}$ (Sugar), $S^{in}_{Bu} = 0$ (Butyrate), $S^{in}_{Pro} = 0$ (Propionate), $S^{in}_{Ac} = 0$ (Acetate), $S^{in}_{CH_4} = 0$ (Methane) (color figure online)

**Fig. 19:**
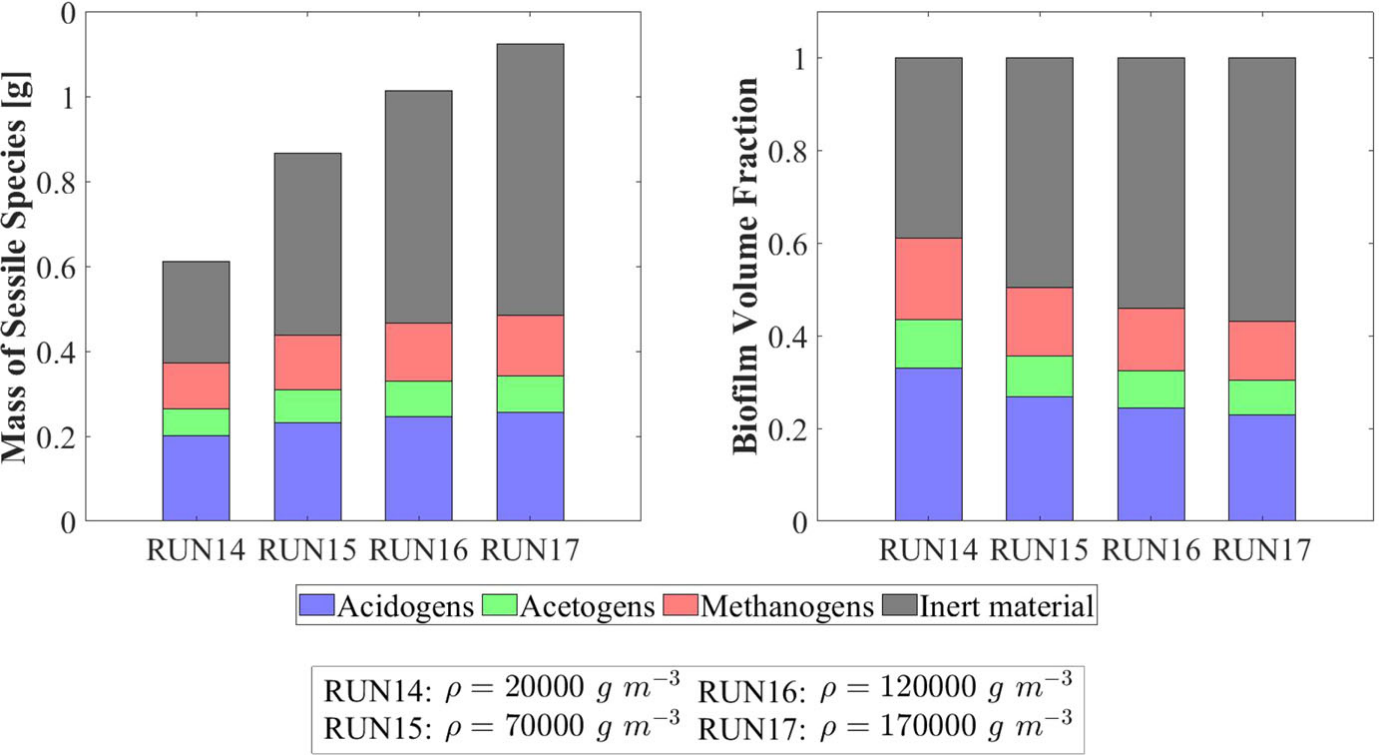
NS4 - Mass (left) and relative abundances (right) of microbial species within the granule at $T = 300 \ d$ for different biomass densities $\rho$. Influent wastewater composition: $S^{in}_{Su} = 3500 \ g \mathrm{m}^{-3}$ (Sugar), $S^{in}_{Bu} = 0$ (Butyrate), $S^{in}_{Pro} = 0$ (Propionate), $S^{in}_{Ac} = 0$ (Acetate), $S^{in}_{CH_4} = 0$ (Methane) (color figure online)

In this context, a numerical study (NS4) is performed to describe the evolution of biofilm granules with different biomass densities. Four simulations (RUN14 - RUN17) are carried out using four different values of biomass density $\rho$ (RUN14: $\rho = 20000 \ g~\ m^{-3}$, RUN15: $\rho = 70000~\mathrm{g\,m}^{-3}$, RUN16: $\rho = 120000~\mathrm{g\,m}^{-3}$, RUN17: $\rho = 170000~\mathrm{g\,m}^{-3}$). The concentration of soluble substrates in the influent wastewater $S^{in}_j$ and the initial concentration of planktonic biomasses within the reactor $\psi ^*_{i,0}$ set for this numerical study are the same used in NS1 and are reported in Table 3. Numerical results are summarized in Figs. 17–19.

The evolution of the granule radius *R*(*t*) over time is shown in Fig. 17. It is clear that the higher the biomass density of the granule, the smaller the steady-state radius achieved. This is due to different mass transfer conditions occurring within the granule: higher biomass densities entail higher fluxes of soluble substrates exchanged between the bulk liquid and granules. For this reason, for higher densities, the substrates in the bulk liquid are consumed faster and the metabolic growth rates driving the growth of the granule are, on average, lower. This leads to smaller granules, in accordance with Liu and Tay (2002).

The microbial ecology for the four values of $\rho$ and at $T = 70 \ d$ is reported in Fig. 18. As can be seen, granules with different densities have different microbial distributions. In particular, when $\rho =20,000 \mathrm{g~m}^{-3}$ (RUN14) an homogeneous distribution is observed: although acidogens (blue) have a tendency to gather in the outermost layers and methanogens (red) and acetogens (green) have the tendency to populate the internal part, the microbial distribution within the granule is fairly homogeneous. This is due to the mass transfer of the soluble substrates within the granule: low biomass density leads to small gradients of soluble substrates across the granule and an homogeneous growth of the different microbial species is observed throughout the biofilm. As the density of the biofilm increases (RUN15–RUN17), the gradient of soluble substrates across the biofilm increases and the layered distribution of the biomass is clearer and more visible: acidogens are strictly confined in the outer layer, while acetogens and methanogens are mostly present in the inner part. These results are in agreement with the experimental evidence reported by Batstone et al. (2004).

In Fig. 19 the mass (left) and the relative abundance (right) of the sessile microbial species within the biofilm are shown at the steady-state condition ($T=300 \ d$). Higher biomass density leads higher amount (left) and fractions (right) of dead biomass accumulated as inert (black).

### NS5—Effects of Erosive Detachment

The evolution of the granule size is the result of a dynamic equilibrium between biomass growth, attachment of new biomass and detachment processes. The detachment flux is essentially related to the erosion process occurring on the granule surface, due to the effect of the hydrodynamic shear forces developing in the bioreactor (Arcand et al. 1994). These forces are highly variable due to the influence of several factors, such as liquid upflow velocity, gas production, particle–particle collision, eventual mixing systems and the geometry of the bioreactor (Liu and Tay 2002; Tay et al. 2001).

Through this perspective, it is interesting to investigate the role of detachment phenomena induced by shear stress on the anaerobic granulation process, and to study their effects on granule size and, consequently, on the distribution, amount and relative abundance of sessile biomass within the granule. For this purpose, the fifth and last study (NS5) is carried out based on eight simulations (RUN18 - RUN25) with eight different values of the detachment coefficient $\lambda$, to simulate different shear stress conditions. The values used are $\lambda = 4, 8, 12, 16, 20, 24, 28, 32 \ \mathrm {m}^{-1} \ d^{-1}$. The concentration of soluble substrates in the influent wastewater $S^{in}_j$ and the initial concentration of planktonic biomasses within the reactor $\psi ^*_{i,0}$ set for this numerical study are the same used in NS1 and are reported in Table 3.

**Fig. 20:**
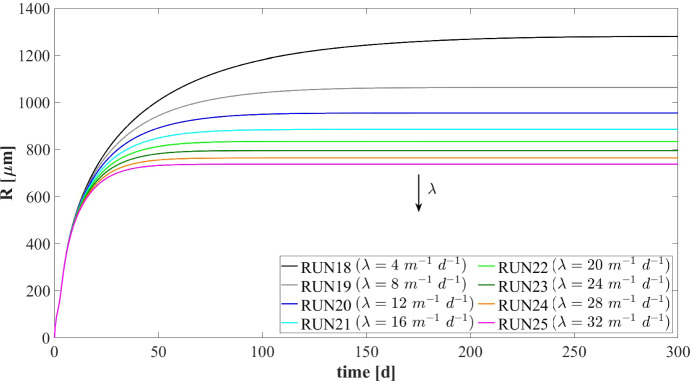
NS5 - Biofilm radius evolution over time for different detachment coefficients $\lambda$. Influent wastewater composition: $S^{in}_{Su} = 3500 \ g~\mathrm{m}^{-3}$ (Sugar), $S^{in}_{Bu} = 0$ (Butyrate), $S^{in}_{Pro} = 0$ (Propionate), $S^{in}_{Ac} = 0$ (Acetate), $S^{in}_{CH_4} = 0$ (Methane) (color figure online)

It should be noted that, contrary to the attachment, the detachment phenomenon increases as the biofilm size increases and has a negligible effect on the initial phase of granulation ($\sigma _d(t)=\lambda R^2(t)$). For this reason, the study does not focus on initial biofilm formation but investigates the long-term effects of the detachment process.

Figure 20 summarizes the effects that different detachment conditions have on the variation of the granule radius *R*(*t*) over time and on the granule steady-state size. As explained above, the formation and the initial evolution of the granule are not affected by the detachment phenomenon. Indeed, it is clear that the trend of *R*(*t*) is not influenced by $\lambda$ until $T = 10$-$20 \ d$. When the granule reaches a $600 \ \mu m$ radius, it becomes very sensitive to the detachment coefficient: as $\lambda$ increases, the erosion phenomenon increases, and a smaller steady-state granule size is achieved. However, the steady-state granule radius has a less than linear behaviour with increasing $\lambda$. Furthermore, in the case of positive attachment flux, steady-state *R*(*t*) tends asymptotically to 0 for $\lambda$ tending towards an infinite value. Indeed, when $R(t) = 0$ the detachment flux is null (see Eq.19) and any positive value of attachment flux is enough to trigger the expansion of the spherical free boundary domain.

**Fig. 21:**
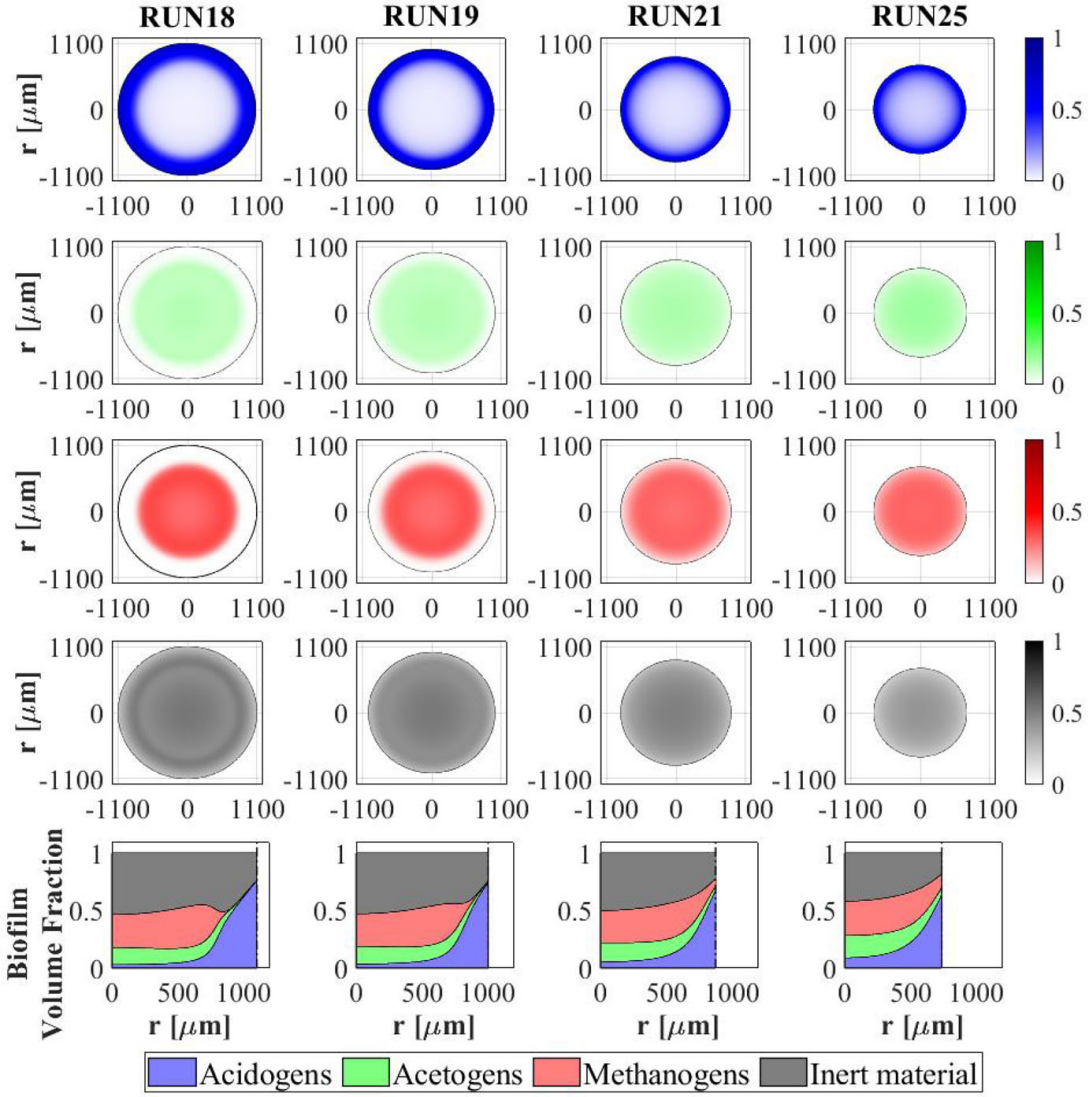
NS5 - Microbial species distribution in the diametrical section and across the radius of the granule at $T = 70 \ d$, for different detachment coefficients. RUN18: $\lambda = 4~\mathrm{m}^{-1} \ d^{-1}$, RUN19: $\lambda = 8~\mathrm{m}^{-1} \ d^{-1}$, RUN21: $\lambda = 16~\ m^{-1} \ d^{-1}$, RUN25: $\lambda = 32 \mathrm{m}^{-1} \ d^{-1}$. Influent wastewater composition: $S^{in}_{Su} = 3500 \ g~\mathrm{m}^{-3}$ (Sugar), $S^{in}_{Bu} = 0$ (Butyrate), $S^{in}_{Pro} = 0$ (Propionate), $S^{in}_{Ac} = 0$ (Acetate), $S^{in}_{CH_4} = 0$ (Methane) (color figure online)

Figure 21 presents the distribution of sessile biomasses within the granule under four different detachment conditions ($\lambda = 4, 8, 16, 32 \mathrm{m}^{-1} \ d^{-1}$), at $T = 70 \ d$. As $\lambda$ increases, an increase in the active biomass fraction $f_{Su}+f_{Bu}+f_{Pro}+f_{Ac}$ and a reduction in the inert material $f_{I}$ occur within the granule. Moreover, for all $\lambda$ values the granule is mainly comprised of acidogens (blue) in the external layers, and methanogens (red) and acetogens (green) at the nucleus. This distribution appears more evident when the granule is larger (low $\lambda$) and there are higher gradients of soluble substrates along the radius.

**Fig. 22:**
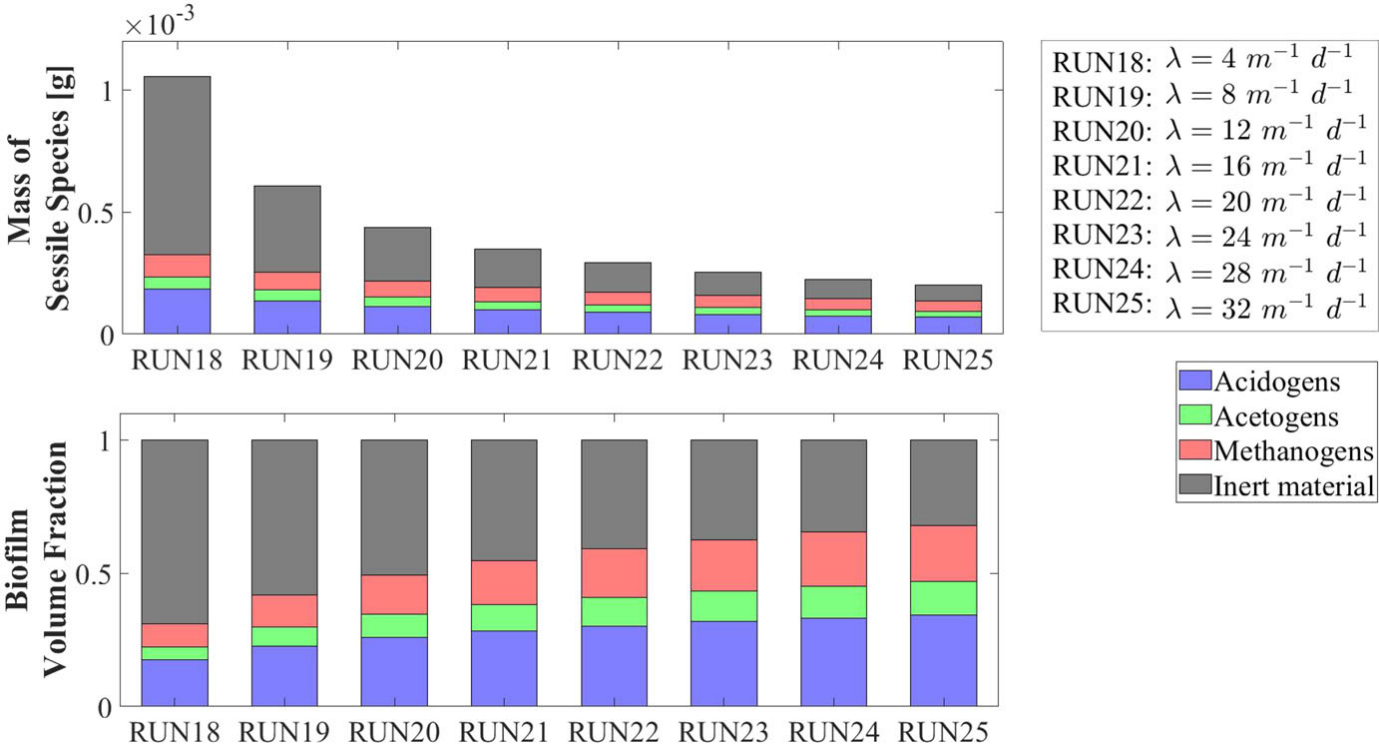
NS5 - Mass (top) and relative abundances (bottom) of microbial species within the granule at $T = 300 \ d$ for different detachment coefficients $\lambda$. Influent wastewater composition: $S^{in}_{Su} = 3500 \ g~\mathrm{m}^{-3}$ (Sugar), $S^{in}_{Bu} = 0$ (Butyrate), $S^{in}_{Pro} = 0$ (Propionate), $S^{in}_{Ac} = 0$ (Acetate), $S^{in}_{CH_4} = 0$ (Methane) (color figure online)

Finally, Fig. 22 reports the steady-state mass (top) and the relative abundance (bottom) of sessile microbial species within the granule under different detachment conditions, at $T=300 \ d$. Since the density is constant and equal in all simulations, the total sessile mass within the granule is directly proportional to the granule size. Then, a higher value of detachment coefficient $\lambda$ leads to a smaller granule, and consequently, a smaller sessile mass both overall and for individual species (Fig. 22-top). The relative abundances shown in Fig. 22 (bottom) confirm the results presented in Fig. 21: higher erosion conditions lead to smaller granules which are characterized by a higher fraction of active biomass.

### NS6—Effects of Number of Granules

**Fig. 23:**
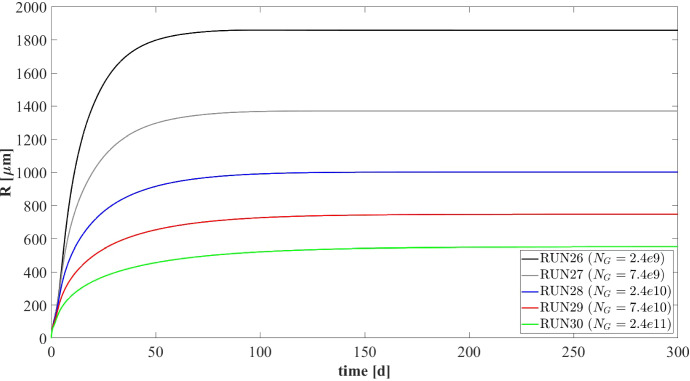
NS6 - Biofilm radius evolution over time for different numbers of granules $N_G$. Influent wastewater composition: $S^{in}_{Su} = 3500 \ g \mathrm{m}^{-3}$ (Sugar), $S^{in}_{Bu} = 0$ (Butyrate), $S^{in}_{Pro} = 0$ (Propionate), $S^{in}_{Ac} = 0$ (Acetate), $S^{in}_{CH_4} = 0$ (Methane) (color figure online)

**Fig. 24:**
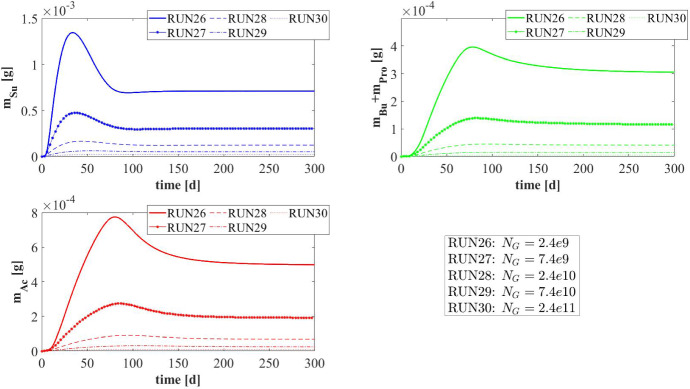
NS6 - Evolution of mass of sessile species within the reactor. $m_{Su}$: mass of sugar fermenters, $m_{Bu}$: mass of butyrate consumers, $m_{Pro}$: mass of propionate consumers, $m_{Ac}$: mass of acetoclastic methanogens. Influent wastewater composition: $S^{in}_{Su} = 3500 \ g \mathrm{m}^{-3}$ (Sugar), $S^{in}_{Bu} = 0$ (Butyrate), $S^{in}_{Pro} = 0$ (Propionate), $S^{in}_{Ac} = 0$ (Acetate), $S^{in}_{CH_4} = 0$ (Methane) (color figure online)

**Fig. 25:**
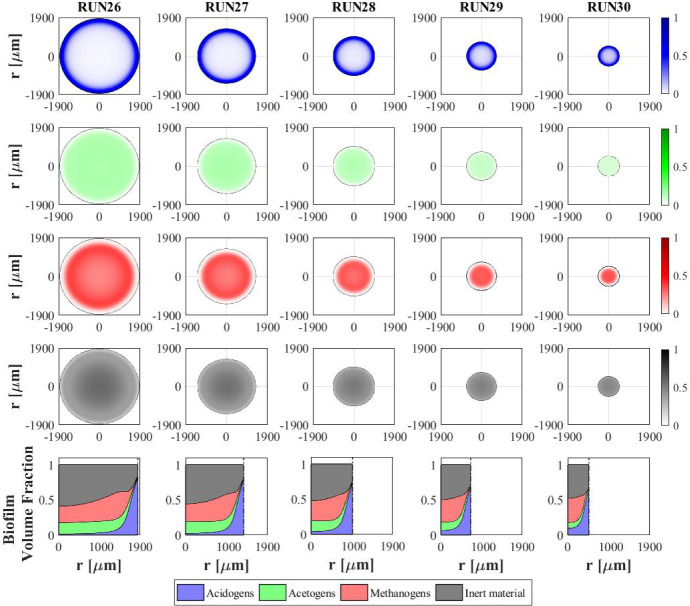
NS6 - Microbial species distribution in the diametrical section and across the radius of the granule at $T = 70 \ d$, for different numbers of granules $N_G$. RUN26: $N_G = 2.4e9$, RUN27: $N_G = 7.4e9$, RUN28: $N_G = 2.4e10$, RUN29: $N_G = 7.4e10$, RUN30: $N_G = 2.4e11$. Influent wastewater composition: $S^{in}_{Su} = 3500 \ g \ m^{-3}$ (Sugar), $S^{in}_{Bu} = 0$ (Butyrate), $S^{in}_{Pro} = 0$ (Propionate), $S^{in}_{Ac} = 0$ (Acetate), $S^{in}_{CH_4} = 0$ (Methane) (color figure online)

**Fig. 26:**
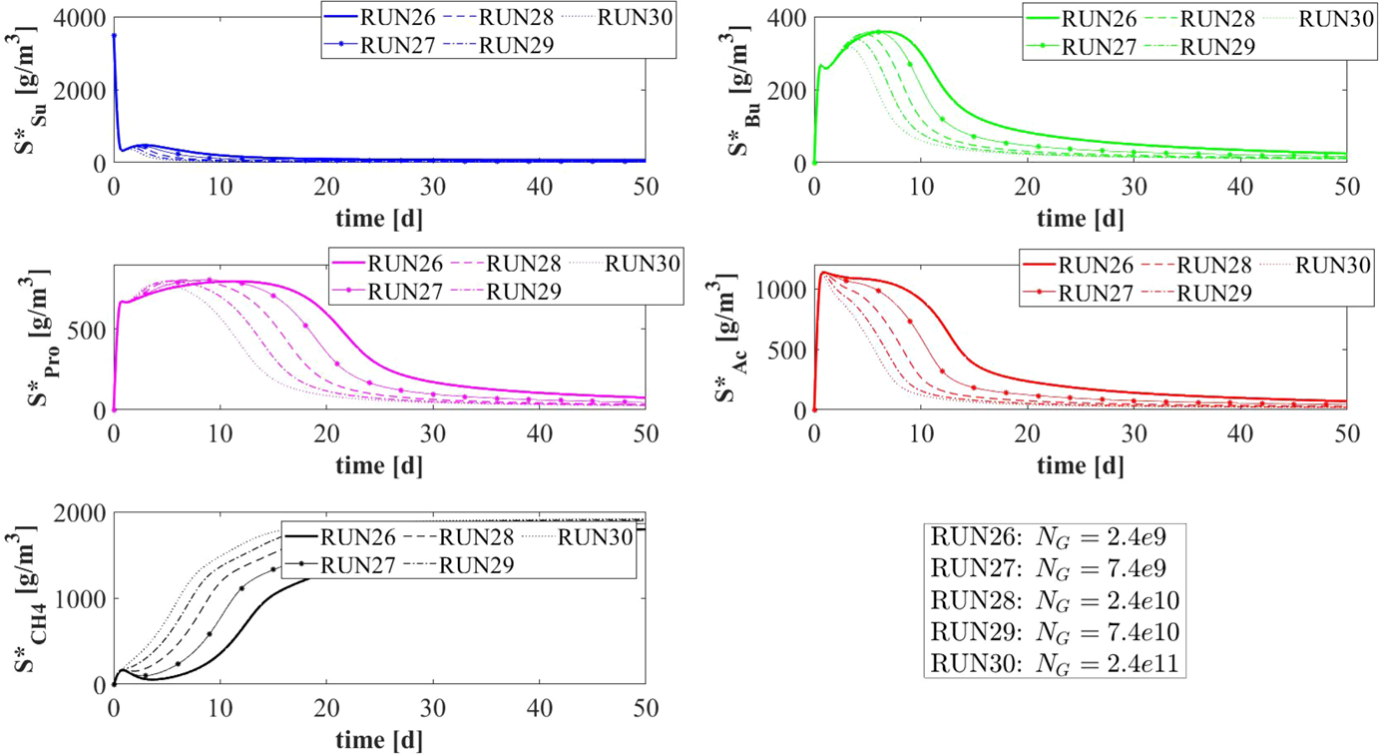
NS6 - Evolution of soluble substrates concentrations within the bulk liquid for different numbers of granules $N_G$. $S^*_{Su}$: Sugar, $S^*_{Bu}$: Butyrate, $S^*_{Pro}$: Propionate, $S^*_{Ac}$: Acetate, $S^*_{CH_4}$: Methane. Influent wastewater composition: $S^{in}_{Su} = 3500 \ g~\mathrm{m}^{-3}$ (Sugar), $S^{in}_{Bu} = 0$ (Butyrate), $S^{in}_{Pro} = 0$ (Propionate), $S^{in}_{Ac} = 0$ (Acetate), $S^{in}_{CH_4} = 0$ (Methane) (color figure online)

**Fig. 27:**
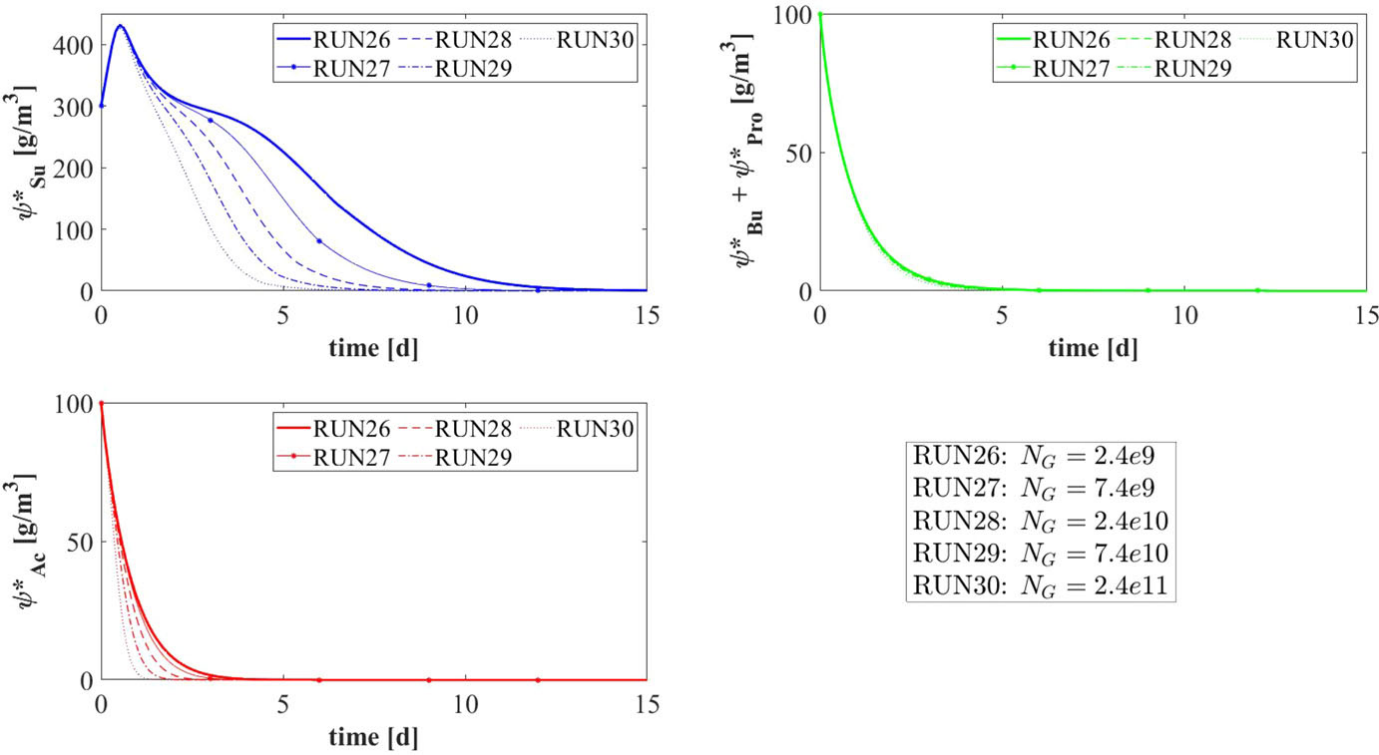
NS6 - Evolution of planktonic biomass concentrations within the bulk liquid for different numbers of granules $N_G$. $\psi ^*_{Su}$: Sugar fermenters, $\psi ^*_{Bu}$: Butyrate consumers, $\psi ^*_{Pro}$: Propionate consumers, $\psi ^*_{Ac}$: Acetoclastic methanogens. Influent wastewater composition: $S^{in}_{Su} = 3500 \ g~\mathrm{m}^{-3}$ (Sugar), $S^{in}_{Bu} = 0$ (Butyrate), $S^{in}_{Pro} = 0$ (Propionate), $S^{in}_{Ac} = 0$ (Acetate), $S^{in}_{CH_4} = 0$ (Methane) (color figure online)

The latest numerical study (NS6) has been developed with the aim of investigating the effects of the parameter $N_G$ (number of granules) on the process. For this purpose, five simulations (RUN26 - RUN30) have been carried out with different values of $N_G$ (RUN26: $N_G = 2.4e9$, RUN27: $N_G = 7.4e9$, RUN28: $N_G = 2.4e10$, RUN29: $N_G = 7.4e10$, RUN30: $N_G = 2.4e11$). The concentration of soluble substrates in the influent wastewater $S^{in}_j$ and the initial concentration of planktonic biomasses within the bioreactor $\psi ^*_{i,0}$ are set equal to NS1 and are reported in Table 3. The results of this study are shown in Figs. 23-27.

Figure 23 shows the evolution of the granule radius over time. The steady-state granule size appears to be strongly affected by $N_G$. In particular, higher $N_G$ leads to smaller granules. This result is intuitive: indeed, the availability of substrates for the single granule reduces when $N_G$ increases, and this leads to a lower metabolic growth of sessile biomass.

Although the size and mass of the single granule decrease as $N_G$ increases, an increase in the overall mass of sessile species within the bioreactor is observed, as shown in Fig. 24. This results in an increase in the filling ratio (biomass volume over reactor volume): going from RUN26 ($N_G = 2.4e9$) to RUN30 ($N_G = 2.4e11$), the filling proportion increases from $16\%$ to $42\%$.

The microbial distribution within the granules for the five values of $N_G$, at $T = 70 \ d$, is reported in Fig. 25. From this figure it is clear that $N_G$ does not significantly influence the microbial stratification of the granules. This occurs because the higher gradient of substrates concentration within the larger granules is balanced by the higher availability of substrates for the single granule (lower $N_G$).

The difference in the trend of sessile masses over time affects the trend of the substrates concentration within the reactor, as shown in Fig. 26. When $N_G$ is higher, sessile masses in the reactor are greater throughout the process and consequently the influent substrate and its products are consumed more rapidly. In addition, small differences are also observed in the equilibrium values: when $N_G$ is lower, the amount of biomass present in the bioreactor is not able to complete the degradation of sugar, butyrate, propionate and acetate, which are present even at the steady-state condition, albeit at small concentrations. This leads to slightly different efficiencies in terms of methane production.

Finally, the overall attachment flux occurring from the bulk liquid to the granules in the first days clearly increases as the number of granules $N_G$ increases. Then, a more rapid reduction in the concentration of planktonic species is observed (Fig. 27).

## Discussion

### Model Assumptions

An anaerobic granular biofilm reactor is an extremely complex and heterogeneous, multiphase, biological system, characterized by properties that vary over time and space. This system is constituted by a liquid medium where an assorted microbial meta-community is immersed. Most of this community is organized into granular aggregates: individual, spherical, biological structures with variable densities and comprised of species representing several different microbial trophic groups in sessile form which enhance the spatial heterogeneity. The life cycle of the de novo granules includes an initial phase of granulation, which leads to granule formation; a maturation phase, in which the granule size increases; and a final phase of breaking (Mills et al. 2021). The pieces of biofilm deriving from the breaking of a granule may, in turn, originate new granules. During this evolution, the granule is affected by complex phenomena, which radically influence its structure and suddenly change its properties: granulation processes of planktonic biomass; metabolic growth and decay of sessile biomass; particle-particle interactions; EPS secretion; gas production; invasion processes by planktonic cells; and detachment processes induced by intense hydrodynamic conditions and shear stress. The combination of these factors leads to a biological community consisting of many granules that co-exist in parallel, and vary, over time, and which differ from one another in size, density and microbial distribution. In addition, the location inside the bioreactor influences the characteristics of the granules. Indeed, due to the geometry and the hydrodynamic conditions of these systems, gradients in the concentrations of soluble substrates establish along the bioreactor and amplify the differences between granules located at different points throughout the system. However, in some cases mixing devices are added to the system, to enhance the circulation of the sludge granules and to reduce the gradients without the need to increase the flow rates and velocity.

Given its physical and biological complexity, the mathematical modelling of an anaerobic granular-based system necessarily requires the introduction of some model assumptions. In this perspective, the model presented in this work describes the anaerobic granular system as a domain having a constant liquid volume where a fixed number of granules is immersed. All granules are assumed to have identical evolution and properties (same size, same density, same constituents). The single granule is modelled as a spherical, free boundary domain having a biomass density constant in time and space. Under the assumption of perfect mixing, the properties of the bulk liquid, in particular the concentration of soluble substrates and planktonic biomass, are supposed to be the same at every point and vary only over time. The attachment is modelled as a continuous, deterministic process which depends linearly on the concentration of the planktonic biomass. The detachment process is modelled as a continuous mass flux that detaches from the granule due to the effect of shear-induced erosion. No contribution by detachment to planktonic or attached biomass is considered in this model. Indeed, the detached biomass has different characteristics from both sessile and planktonic biomass and needs several hours to return to the planktonic state (Rollet et al. 2009; Berlanga et al. 2014; Rumbaugh and Sauer 2020). Since the HRT of this bioreactor is very low, the detached biomass does not have enough time to convert. Anyway, modelling the detached species within the bioreactor as a new set of variables, able to grow and convert the soluble substrates, was found to have a negligible effect on the biological dynamics involved (data not shown). The process of invasion by planktonic cells present in the bulk liquid is modelled as a diffusive transport across the granule. This eliminates the restrictions on the granule’s ecological structure that could be generated in particular cases, and guarantees the growth of each sessile microbial species where optimal metabolic conditions prevail. Finally, suspended substrates in the influent wastewater, gas transfer processes and EPS production are neglected as they are not significant for the purposes of the numerical investigation presented in this work: qualitatively studying the anaerobic granulation process, the evolution of the granules over time and the related ecological succession.

It is emphasized that all assumptions introduced are consistent and do not compromise the objectives of the model: describing the genesis, evolution and ecology of anaerobic granules and the biological treatment process of an anaerobic granular-based bioreactor.

### Size, Microbial Distribution and Ecology of Biofilm Granules, and the Evolution of Bulk Liquid Characteristics

The dimensional evolution of the granule is described through the expansion of a spherical, free boundary domain, whose radius varies over time. In particular, the de novo granulation process is modelled starting from an initial condition where all the biomass present in the reactor is in planktonic form and there are no biofilm granules. Subsequently, the aggregation of planktonic cells leads to granule formation. The evolution of the granule over time is governed by the positive contributions of sessile metabolic growth and attachment flux of planktonic biomass and by the negative contribution of the erosive detachment flux. In the initial phase of the process, the size of the granule grows exponentially due to the high availability of substrates. This leads to high rates of sessile metabolic growth, and the high attachment flux induced by the presence of planktonic biomass within the bulk liquid and the negligible detachment flux, proportional to the granule size ($\sigma _d(t)=\lambda R^2(t)$). Later, the concentrations of soluble substrates and the planktonic biomass within the bulk liquid reduce. This leads to a reduction in metabolic growth and attachment flux. In addition, the detachment flux intensifies as a result of the granule size increase. Consequently, the growth of the granule decreases until it reaches a steady-state value regulated by the balance between the positive source from sessile growth and the negative detachment flux.

The numerical studies show that the evolution of the granule size and its equilibrium value are deeply influenced by some factors, such as the erosion intensity, the mass transfer of soluble substrates, the composition of the influent, the granulation properties of the planktonic biomass present in the inoculum and the number of granules within the system. The model results presented in NS5 report that the granule size is governed by the erosive detachment: intense detachment forces on the granule surface limits its growth. This qualitative result is in agreement with previous observations (Liu and Tay 2002; Tay et al. 2001; Arcand et al. 1994).

As reported in NS2, the composition of the influent can affect the size of the granule due to the anabolic pathway of the species involved in the process. For example, the consumption of sugar induces more growth of sessile biomass compared to the consumption of the other substrates. As a result, for equal OLRs, an influent with higher sugar concentrations leads to the growth of more sessile biomass and larger granules. NS4 reports that more soluble substrate is consumed in denser granules, per unit of volume, and that the metabolic growth rates therefore decrease faster and lead to smaller equilibrium sizes, in accordance with Liu and Tay (2002). NS3 suggests that the granulation properties of the planktonic biomass regulate the evolution of the granule, especially in the initial phase of biofilm formation. More precisely, when the planktonic biomass is more prone to form biofilm structures and to grow in sessile form, the granulation process is more intense, and the granules grow faster. Finally, NS6 shows that the number of granules present in the system affects the steady-state granule size by influencing the substrates dynamics within the bulk liquid.

In addition, the model describes the microbial ecology that develops in the granule and shows the distribution of the different microbial species. In the initial phase, the granulation process is governed by acidogenic and methanogenic species. This is in agreement with several studies (Trego et al. 2020b; Pol et al. 2004; Jian and Shi-yi 1993) reporting that methanogens play a fundamental role in the formation of the initial nucleus of the granule. Indeed, some methanogenic cells have a filamentous morphology (Trego et al. 2020b) and the ability to employ quorum sensing strategies (Li et al. 2015; Zhang et al. 2012; Li et al. 2014) to improve their granulating properties and increase granulation efficiency. At the same time, acidogens have higher growth rates than other species and are therefore abundant in the granule from the beginning of the process. Acidogens and methanogens exhibit the tendency to grow in different areas of the granule: the first in the outermost layer and the second in the inner part. This distribution becomes more evident over time. In particular, when the granule is mature, it is constituted by a large internal part populated by methanogens shielded by a thin external layer of acidogens. This happens because the growth rate of acidogens is higher than other species in the presence of adequate concentrations of substrate (external layers), while methanogens are able to adapt better than other species to shortage of substrates (internal layers). For extended periods, an homogeneous growth of acetogens, and the presence of inert material deriving from biomass decay, is also observed. Many experimental studies (Sekiguchi et al. 1999; Batstone et al. 2004) show a microbial distribution within anaerobic granules similar to the results proposed by the model.

However, the model suggests that some factors, such as the composition of the influent, the biomass density, and the erosive detachment intensity can radically affect the microbial distribution. For example, NS2 shows that in the case of higher sugar concentrations within the influent wastewater, the granules that develop during the process have more acidogenic biomass. Conversely, when only VFAs are present in the influent, acidogens do not develop, and methanogens and acetogens dominate the granule. Furthermore, the model qualitatively reproduces the microbial distribution observed by Batstone et al. (2004) in granules of different densities (study NS4). The biomass density deeply influences the mass transfer of the soluble substrates and, thus, the microbial ecology. Accordingly, low densities lead to small gradients of soluble substrates across the granule, and an homogeneous distribution of the microbial species is observed throughout the biofilm. More pronounced gradients develop within denser granules, leading to a more stratified distribution of biomass. According to the NS5 study, the detachment intensity appears to be an additional factor affecting the microbial distribution in an anaerobic granule, especially affecting the amount of active and inactive biomass: as mentioned above, intense erosion results in the formation of small granules where there is high availability of substrates, and active biomass prevails over inactive biomass; meanwhile, weaker erosion induces the formation of larger granules populated by more inactive biomass accumulating in the innermost zone of the granule due to the limitation of soluble substrates.

Furthermore, the model describes how the granulation process influences the characteristics of the bulk liquid and the effluent, in particular the concentration of planktonic biomasses and soluble substrates. The planktonic biomasses are present in the inoculum initially introduced in the bioreactor and represent the microbial community that initiates the granulation process. In the first few days, the concentration of planktonic biomass decreases rapidly as a consequence of attachment and dilution. The first concerns the aggregation of planktonic biomass, which converts to sessile form and contributes to the establishment of granular structures. The second is the result of the short HRT of the bioreactor. Indeed, as already reported, the HRT of granular-based systems is fixed short enough to guarantee hydrodynamic conditions and shear forces optimal for the granulation process. Such HRTs are highly unfavourable for planktonic biomass, which has insufficient time to grow and is consequently diluted (Pol et al. 2004). In agreement with this, after a few days the planktonic biomass is completely washed out. Planktonic acidogens remain in the bulk liquid longer than other species due to their higher growth rate. Soluble substrates are produced, or consumed, in the bulk liquid due to the effect of planktonic and sessile biomass. In the initial phase, the granules are small and the substrates are converted mainly by planktonic biomass. Subsequently, when the size of the granules increases, and the planktonic biomass has already been washed out, the trend of the substrates in the bulk liquid is governed by the granular biomass. Note that soluble substrates and planktonic biomass achieve the steady-state values over a shorter time than sessile biomass in the granule. The effluent appears to be purified at the steady-state, with the complete conversion of sugar and VFAs into methane.

Obviously, the trend of planktonic biomass and soluble substrates within the bulk liquid is influenced by the influent composition (study NS2). Furthermore, NS3 shows that the granulation properties of the planktonic microbial community initially present in the bioreactor deeply affect the velocity of the de novo granulation process. When the granulation process occurs faster, the conversion rate of substrates is higher, and the process reaches the steady-state sooner. Accordingly, the model results confirm that improving granulation properties of the microbial community allows for the process to be expedited and long bioreactor start-up times (Li et al. 2015) be reduced, which represents a critical issue in the operation of granular-based systems. Finally, the number of granules present in the bioreactor influences both the dynamics of the planktonic species and the bioreactor performance (NS6). A higher number of granules implies an overall higher attachment flux and, therefore, a faster reduction of planktonic species. At the same time, a higher number of granules leads to higher sessile masses and, consequently, to faster and more effective conversion of sugars and VFAs into biomethane.

## Conclusions

In this work, a mathematical model able to reproduce the de novo granulation process involved in a generic, granular biofilm system has been introduced. The work presents the derivation of the model equations, which govern the expansion of the granule; the growth of sessile biomass; and the transport of substrates and planktonic cells within the granule, under the assumption of radial symmetry. Such equations have been derived from mass balances considerations in the framework of continuum mechanics. Processes of growth, and decay, of attached and planktonic biomass; attachment from bulk liquid to biofilm; detachment from biofilm to bulk liquid; invasion of planktonic cells; and conversion, and diffusion, of soluble substrates are modelled. The model has been applied to anaerobic granular systems to test its qualitative behaviour and study the process for a case of biological and engineering interest. The model takes into account the different contributions that individual microbial trophic groups can provide to the granulation process. Specifically, in the case of the anaerobic digestion pathway, the model is able to consider the fundamental role that some species of methanogens play in granulation by setting appropriate values of attachment velocity. The results shown describe exhaustively the anaerobic granulation process; the main properties of the granules, such as dimensional evolution, ecology, biomass distribution, microbial relative abundance and distribution of soluble substrates; and the evolution of the bulk liquid characteristics (soluble substrates and planktonic biomass). Finally, further numerical studies have been carried out to investigate the effects on the process of key factors.

The most interesting observations resulting from the numerical studies include:

- The anaerobic granule presents a typical microbial stratification: methanogens and acetogens populate the innermost layers of the granule and are shielded by a thin external layer of acidogens; the thickness of these layers depends on multiple factors (e.g. composition of the influent wastewater, biomass density, detachment forces).
- Intense flow rates and short HRTs, typical of granular biofilm systems, limit the growth of planktonic biomass, which is washed out.
- The influent wastewater composition affects the evolution, ecology and microbial stratification of the granules.
- The granulation properties of the planktonic biomass considerably influence the start-up period of the system. Strategies, such as controlling hydrodynamic conditions, stimulating quorum sensing stimulation and bioaugmentation may reduce the duration of this stage and enhance the efficiency of bioreactor start-up.
- The density of biomass regulates the mass transfer of soluble substrates and, consequently, the distribution of microorganisms within the granule: denser granules have a more layered structure whilst less dense granules have a more homogeneous structure.
- The detachment erosion has a large impact on the granule size and the ratio of active to inactive biomass: more erosion leads to smaller granules constituted by larger fractions of active biomass.
- The number of granules can significantly affect the granulation process (with respect to granule size) and slightly impact on bioreactor performance (with respect to substrate conversion and methane production).

Most of the results shown are qualitatively in accordance with the experimental evidence from the literature. Accordingly, this model is able to correctly simulate both the formation and maturation of anaerobic granules by focusing on both the transient and the steady state. From an engineering point of view, this allow us to conclude that the model proves to be a useful tool in studying both the start-up and the routine treatment processes of anaerobic granular biofilm systems. Furthermore, the model can be applied to any biological process proceeding in a granular-based system by choosing suitable model variables and kinetic expressions.

In any case, some model parameters, such as the values of the attachment velocities of the planktonic biomass, are introduced here for the first time and should be calibrated, and validated, on the basis of experimental data. Finally, with a view to future work, the detachment process leading to the breaking of granules and the consequent formation of further, new granules, could be included in the model, to describe the entire life cycle of biofilm granules.
